# Towards highly efficient NIR II response up-conversion phosphor enabled by long lifetimes of Er^3+^

**DOI:** 10.1038/s41467-022-34350-1

**Published:** 2022-11-01

**Authors:** Xiumei Yin, Wen Xu, Ge Zhu, Yanan Ji, Qi Xiao, Xinyao Dong, Ming He, Baosheng Cao, Na Zhou, Xixian Luo, Lin Guo, Bin Dong

**Affiliations:** 1 School of Physics and Materials Engineering, Dalian Minzu University, 18 Liaohe West Road, Dalian, 116600 China; 2 School of Chemistry and Environment, Beijing University of Aeronautics & Astronautics, 37 Xueyuan Road, Beijing, 100191 China

**Keywords:** Optical materials and structures, Materials for optics

## Abstract

The second near-infrared (NIR II) response photon up-conversion (UC) materials show great application prospects in the fields of biology and optical communication. However, it is still an enormous challenge to obtain efficient NIR II response materials. Herein, we develop a series of Er^3+^ doped ternary sulfides phosphors with highly efficient UC emissions under 1532 nm irradiation. β-NaYS_2_:Er^3+^ achieves a visible UC efficiency as high as 2.6%, along with high brightness, spectral stability of lights illumination and temperature. Such efficient UC is dominated by excited state absorption, accompanied by the advantage of long lifetimes (^4^I_9/2_, 9.24 ms; ^4^I_13/2_, 30.27 ms) of excited state levels of Er^3+^, instead of the well-recognized energy transfer UC between sensitizer and activator. NaYS_2_:Er^3+^ phosphors are further developed for high-performance underwater communication and narrowband NIR photodetectors. Our findings suggest a novel approach for developing NIR II response UC materials, and simulate new applications, eg., simultaneous NIR and visible optical communication.

## Abstract (web summary)

Development of efficient upconversion (UC) phosphors that emit under irradiation in the NIR II region is challenging - most UC materials rely on the presence of sensitizers absorbing at shorter wavelengths. Here, authors synthesize Er^3+^ doped ternary sulfides phosphors with visible UC efficiency up to 2.6% and long emission lifetimes under 1532 nm irradiation, via an excited state absorption mechanism.

## Introduction

Lanthanide doped photon up-conversion (UC) materials can absorb two or more low-energy photons, and emit high-energy photons, which have attracted extensive attention due to the superior optical performance, such as large anti-Stokes shift, excellent stability against photo-bleaching, and no auto-fluorescence^1–5^. The first discovery of an effective UC host material (NaYF_4_) was made by Pierce in 1972^6^. Until 2004, the hexagonal phase NaYF_4_ (β-NaYF_4_) with high UC efficiency was demonstrated^7^. With the development of nanotechnology, lanthanide doped UC materials experienced the explosive growth, especially in biological applications^8–10^, photon-conversion devices^11,12^, super-resolution nanoscopy^13–15^, and information storage and security^16–18^. Much efforts have been devoted to manipulate UC, and Yb^3+^, Nd^3+_^sensitized energy transfer (ET) and interfacial energy transfer (IET) are recognized as the promising ways to achieve efficient UC luminescence^19–24^.

The near-infrared second spectral region (NIR II), especially for 1530 nm, shows great potential with respect to the information storage and security, 3D display, and high-resolution imaging, etc^25–28^. Particularly, 1530 nm is also the wavelength of optical communication, exhibits a low loss in transmission, and is widely used in metro, long-distance, ultra-long-distance, and submarine optical cable systems^29–32^. However, the development of the highly efficient NIR II 1530 nm response photon UC materials still faces great challenges. Up to date, the recognized efficient UC materials (eg., NaYF_4_:Yb^3+^, Er^3+^/Tm^3+^) are based on the sensitized ET, owing to the efficient ET process from sensitizers (eg., Yb^3+^, Nd^3+^) to activators (eg., Er^3+^, Tm^3+^, Ho^3+^). Despite the great progress made in UC, the existing UC materials strongly depend on the excitation of short near-infrared wavelengths (such as 808 and 980 nm). Er^3+^ has a well spectral response around 1530 nm with a relatively large absorption cross-section, but it is not suitable for sensitizer owing to the lack of matchable activators. A number of reports has announced Er^3+^ doped UC materials pumping around 1530 nm realized via the excited state absorption process of Er^3+^ themselves^33–39^. Disappointedly, their up-conversion quantum yields (UCQYs) are low, less than 0.5%, limited by the deleterious quenching interactions, and strong electron-phonon coupling etc. To date, because of the lack of appropriate host materials, it still remains a challenge to achieve efficient NIR II 1530 nm response UC.

Ternary rare rarth sulfide (MLnS_2_; where M is an alkali metal ion, and Ln is a rare earth ion) materials process the layer structure, low unit cell symmetry, strong covalence and low phonon energy^40^, which are promising materials for efficient NIR II response UC. It was discovered in 1970s^41^, and Güdel et al. reported the UC emissions with UCQY of 21% in NaYS_2_:Er^3+^ crystals under the excitation of 980 nm, but this was obtained at a high incident power of 0.79 kW cm^−2^ at low temperature of 12 K^42^. Since then, little studies have paid attention to these materials.

In this work, we fabricated a series of efficient NIR II 1532 nm response UC materials: MLnS_2_:Er^3+^ (M = Li, Na, K; Ln = Y, Lu, La, Gd). They exhibit cubic (α-) and trigonal (β-) phases depending on the radius ratios between the trivalent lanthanide ions and the monovalent alkali metal ions (RLn^3+^/RM^+^). It should be highlighted that β-NaYS_2_ presents a particularly high efficiency UC emissions with the UCQY as high as 2.6% under 1532 nm excitation (~4.5 W cm^−2^), originating from the exceptionally long lifetimes (^4^I_9/2_, 9.24 ms; ^4^I_13/2_, 30.27 ms) of excited state levels of Er^3+^. Compared to the commercial NaYF_4_:Yb^3+^, Er^3+^ phosphor, the NaYS_2_:Er^3+^ owns much higher UCQY, brightness, and spectral stability of illumination and temperature under the same conditions. Then, the NaYS_2_:Er^3+^ phosphors were employed to achieve multiband responsive NIR photodetectors (800 nm, 980 nm, and 1532 nm), and green light underwater communication.

## Results

### Synthesis and structure characterization of the MLnS_2_:Er^3+^ phosphors

**Fig. 1:**
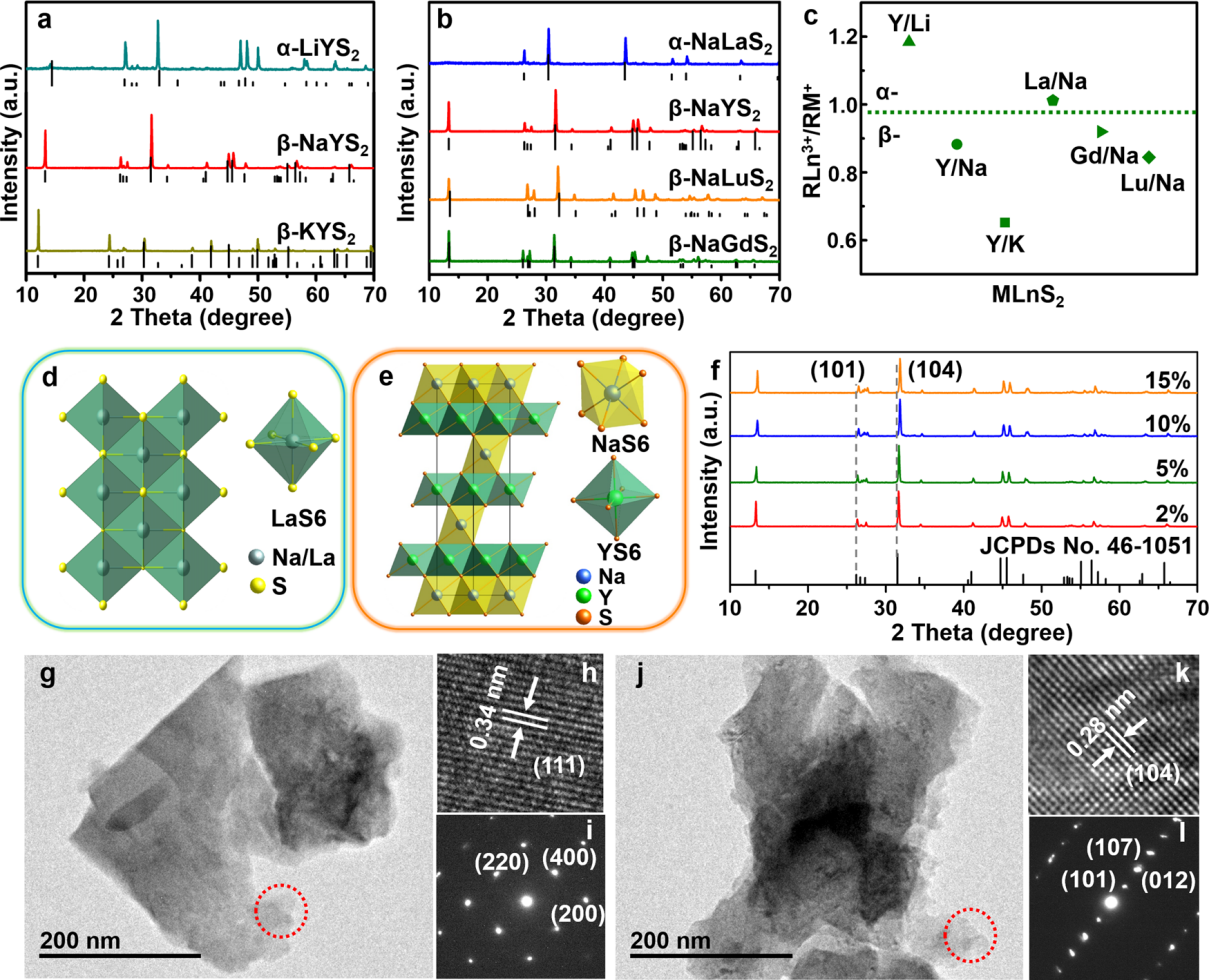
Structure and morphology characterizations of MLnS_2_:Er^3+^. **a**, **b** XRD patterns of MYS_2_:Er^3+^ (M = Li, Na, K) and NaLnS_2_:Er^3+^ (Ln = La, Y, Lu, Gd) prepared at 1173 K. **c** Radius ratios between the trivalent lanthanide ion and the monovalent alkali metal ion (RLn^3+^/RM^+^) of MLnS_2_ (M = Li, Na, K; Ln = La, Y, Lu, Gd), the cubic and trigonal phases are divided by the green dotted line. **d**, **e** Crystal structure models of cubic NaLaS_2_ (α-NaLaS_2_) and trigonal NaYS_2_ (β-NaYS_2_). **f** XRD patterns of β-NaYS_2_ doped with 2, 5, 10, 15 mol% Er^3+^. TEM, HRTEM images, and corresponding SAED of **g–i** α-NaLaS_2_ and **j–l** β-NaYS_2_. Source data is provided in this paper.

A series of MLnS_2_:Er^3+^ UC phosphors were synthesized by the gas-solid reaction method, in which the IA alkali metal elements (M = Li, Na, or K) and trivalent rare earth elements (Ln = Y, Lu, La, or Gd) were selected to occupy the M and Ln sites, respectively (See Methods). Figures 1a and 1b display the X-ray diffraction (XRD) patterns of MYS_2_ and NaLnS_2_ prepared at 1173 K, which coincide well with the corresponding standard cards. The XRD patterns and UC luminescence spectra further reveal that the optimized temperature for synthesis is 1173 K, otherwise the impurity phase of Y_2_O_2_S is formed at 1273 and 1373 K (Supplementary Figs. 3, 4), which leads to the decrease of UC luminescence. Interestingly, MLnS_2_ phosphors have two structural types: NaLaS_2_ and LiYS_2_ possess cubic structure (α-), while KYS_2_, NaGdS_2_, NaLuS_2_, and NaYS_2_ are indexed as trigonal phase (β-) under the same conditions (Fig. 1a, b). As represented in Fig. 1c, the phase structure of MLnS_2_ phosphors mainly depends on the radius ratios between the trivalent lanthanide ions and the monovalent alkali metal ions (RLn^3+^/RM^+^)^43^. When the RLn^3+^/RM^+^ ratio is larger than 1.0, MLnS_2_ tends to form cubic phase (NaCl or Th_3_P_4_ type). Conversely, the β-phase structure is dominant. The α-NaLaS_2_ and β-NaYS_2_ were taken as examples to illustrate the representative structure of MLnS_2_ family (Fig. 1d, e). In α-NaLaS_2_ with cubic structure, Na^+^ and La^3+^ are coordinated with six S^2−^ to form (Na/La-S)6 octahedrons^44^. In β-NaYS_2_, both Na^+^ and Y^3+^ are six-fold coordinated by S^2−^ in form of a regular octahedron and contain empty voids on the special 3a and 3b sites, respectively. The S^2−^ are surrounded by three Na^+^ and three Y^3+^. Both MS6 and LnS6 distorted octahedra are more precisely described as trigonal anti-prisms with centro-symmetric D3d symmetry, where all six bonds M–S in the octahedron possess identical length (as in Oh symmetry)^45,46^. Therefore, Na^+^ and Y^3+^ are orderly situated in alternating NaS6 and YS6 octahedral layers.

In addition, the XRD patterns of NaYS_2_ with various doping concentration of Er^3+^ are recorded in Fig. 1f. β-NaYS_2_ is obtained according to the standard cards (JCPDS No. 46-1051), and two characteristic peaks located at 26.34^°^ and 31.64^°^ correspond to the (101) and (104) crystal planes, respectively. The diffraction peaks of NaYS_2_:Er^3+^ shift to large angle on increasing the doping concentration of Er^3+^ owing to the replacement of Y^3+^ (89.3 pm) by Er^3+^ (88.1 pm) with a smaller radius. The similar morphologies of MLnS_2_:Er^3+^ are obtained as displayed in Supplementary Fig. 5. The transmission electron microscopic (TEM) and high resolution TEM (HRTEM) images of the NaLaS_2_:Er^3+^ and NaYS_2_:Er^3+^ phosphors are presented in Fig. 1g, h and Fig. 1j, k, which are irregular morphology with micron size (Supplementary Fig. 5). The lattice fringes of 0.34 nm and 0.28 nm are associated with (111) and (104) planes of NaLaS_2_ (d-spacing of 0.339 nm; JCPDS No. 38-1391) and NaYS_2_ (d-spacing of 0.282 nm; JCPDS No. 46-1051), respectively. The selected area electron diffraction (SAED) patterns (Fig. 1i, l) further demonstrate the good crystallinity of samples. All of the above results indicate that the MLnS_2_:Er^3+^ phosphors with two phases are successfully fabricated.

### UC luminescence properties of MLnS_2_:Er^3+^

**Fig. 2:**
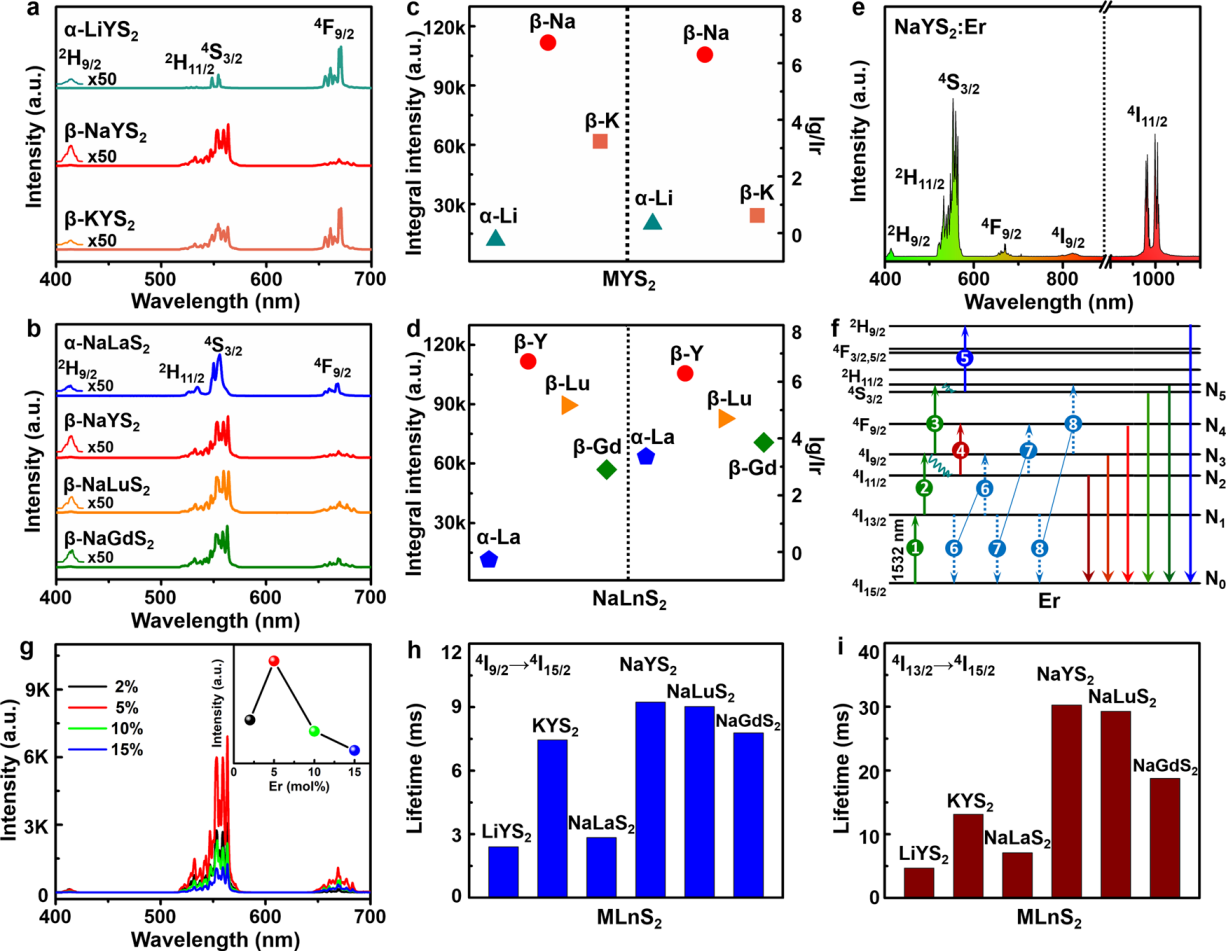
UC luminescence characterization of MLnS_2_:Er^3+^. **a**, **b** Normalized visible UC luminescence spectra of MYS_2_:Er^3+^ (M = Li, Na, K) and NaLnS_2_:Er^3+^ (Ln = La, Y, Lu, Gd) under 1532 nm excitation. **c**, **d** Integral luminescence intensity and green to red emission ratio (Ig/Ir) of MYS_2_:Er^3+^ (M = Li, Na, K) and NaLnS_2_:Er^3+^ (Ln = La, Y, Lu, Gd). **e** UC luminescence spectrum of NaYS_2_:Er^3+^ in visible and NIR regions. **f** UC luminescence populating mechanism of NaYS_2_:Er^3+^ under 1532 nm excitation, the 1-5 present the excited state absorption (ESA) process, 6-8 present the cross-relaxation process. **g** UC luminescence spectra of NaYS_2_ doped with 2, 5, 10, 15 mol% Er^3+^ (inset: integral intensity varies with concentration of Er^3+^). **h-i**, Lifetimes of ^4^I_9/2_→^4^I_15/2_ and ^4^I_13/2_→^4^I_15/2_ transitions of Er^3+^ in MLnS_2_:Er^3+^. Source data is provided in this paper.

The NIR II response UC luminescence characteristics of MLnS_2_:Er^3+^ under 1532 nm excitation are systematically evaluated. As presented in Fig. 2a, b, the blue, green, and red luminescence regions are identified in the normalized visible UC spectra of MLnS_2_:Er^3+^, assigned to the ^2^H_9/2_ → ^4^I_15/2_, ^4^S_3/2_/^2^H_11/2_ → ^4^I_15/2_, and ^4^F_9/2_ → ^4^I_15/2_ transitions of Er^3+^, respectively. Note that, β-MLnS_2_:Er^3+^ show clearer and sharper splitting of emission lines than those of α-MLnS_2_:Er^3+^, implying the better optical performance of β-phosphors. As expected in Fig. 2c, d, the emission intensities of β-MYS_2_:Er^3+^ and β-NaLnS_2_:Er^3+^ are significantly higher than those of α- phase phosphors. NaYS_2_:Er^3+^ is found to be the most efficient UC phosphor in the MLnS_2_:Er^3+^ series under 1532 nm excitation.

The UC intensity ratios of green-to-red emissions in β-MLnS_2_:Er^3+^ are larger compared to the cubic phase ones. These suggest that the β-MLnS_2_:Er^3+^ phosphors are benefit to the UC emissions. The Raman spectra of the MLnS_2_ hosts in Supplementary Fig. 6 demonstrate that the phonon energies of all the samples are lower than 300 cm^−1^. Based on the theory of multi-phonon relaxation, the low-frequency phonon should rarely take contributions of nonradiative relaxation^47^. The phonon energies of all the MLnS_2_ hosts locate within 260–280 cm^−1^, consistently with the reported results^48^. Considering the similar phonon energies of MLnS_2_ hosts, such phenomenon can be explained by the following reason: the Er^3+^ in β-MLnS_2_ are ordered separated by NaS6 octahedral layers along the c axis direction, which blocks the cross relaxation between each other to a certain extent. However, the Er^3+^ in α-MLnS_2_ are randomly disordered on an identical lattice site, inevitably leading to the intensified negative energy exchange^49,50^. The comparison of UC emission intensity between α-NaLaS_2_:0.1–15%Er^3+^ and β-NaYS_2_:0.1–15%Er^3+^ (Supplementary Fig. 7) further proves the above deduction. In addition, the distance of the nearest Y^3+^ (dY-Y) in NaYS_2_ is determined to be 3.9808 Å. Since the probability of energy transfer between luminous centers (concentration quenching) is proportional to 1/d6, the large adjacent distance d often causes a high doping level and intense luminescence. Crystal structural data for NaYS_2_ and NaYF_4_ are summarized in Supplementary Table 1. The excellent characteristics of NaYS_2_ compared to NaYF_4_ are mainly ascribed to the layer structure, lower unit cell symmetry (rhombohedral vs hexagonal), longer minimum Y^3+^ separation distance, stronger covalence, and lower phonon energy. These advantages of NaYS_2_ all contribute to the efficient UC of NaYS_2_:Er^3+^.

The visible-NIR UC luminescence of NaYS_2_:Er^3+^ illuminated at 1532 nm was further investigated to clarify the populating process. Five UC emission bands spanning from 400–1100 nm are given in Fig. 2e. The NIR emission bands in the range of 790 to ~840 nm and 950 to ~1050 nm are attributed to the ^4^I_9/2_, ^4^I_11/2_ → ^4^I_15/2_ transitions of Er^3+^, respectively. A schematic illustration of populating mechanism for NaYS_2_:Er^3+^ pumping under 1532 nm is provided in Fig. 2f, originating from the multi-photon excited state absorption (ESA) process. Firstly, the electrons in the ground state (^4^I_15/2_) of Er^3+^ are pumped to ^4^I_13/2,_ and further excited to ^4^I_9/2_ via resonant illumination, generating ^4^I_9/2_ → ^4^I_15/2_ transition. The electrons on ^4^I_9/2_ jump to the higher excited levels (^4^S_3/2_/^2^H_11/2_) through continuous three-photon absorption, realizing the green emissions. A fraction of electrons on ^4^I_9/2_ decays non-radiatively to ^4^I_11/2_, then populates the ^4^F_9/2_ state and thereby generating ^4^I_11/2_ → ^4^I_15/2_ and red (^4^F_9/2_ → ^4^I_15/2_) emissions, respectively. ET-sensitized UC is always considered as the most efficient strategy to achieve UC emissions, while the excited state absorption is basically limited by the weak absorption cross-section. However, the efficient UC emissions in MLnS_2_:Er^3+^ are realized through an ESA process. It is different from the UC from typical energy transfer systems containing Yb^3+^, Er^3+^, where Yb^3+^ serves as a sensitizer and Er^3+^ as an activator. Thus, the visible and NIR emissions are ascribed to three- and two-photon UCs, as evidenced by the power dependence of the integral UC emission intensity of NaYS_2_:Er^3+^ (Supplementary Fig. 8).

As displayed in Fig. 2g, the visible UC luminescence intensity of NaYS_2_:Er^3+^ initially increases, then decreases with increasing the doping concentration of Er^3+^, in which the optimal doping concentration of Er^3+^ is 5 mol%. Similarly, the corresponding decay time curves and constants of Er^3+^ in Supplementary Figs. 9–13 and Supplementary Table 2 present that the lifetime first increases, and then decreases as the Er^3+^ concentration continuously increases to 15 mol%. Such increase of decay time constants may be attributed to the re-absorption induced by high doping concentration of Er^3+^. A further decrease results from concentration quenching. It should be highlighted that the decay profiles of ^4^S_3/2_ → ^4^I_15/2_ transition (green emissions) change from single exponential to double exponential, suggesting that cooperative UC among Er^3+^ is involved. While they decrease for ^4^I_9/2_ → ^4^I_15/2_ (800 nm), ^4^I_11/2_ → ^4^I_15/2_ (1000 nm), and ^4^I_13/2_ → ^4^I_15/2_ (1500 nm) on increasing the Er^3+^ doping concentration from 2 to 15 mol%. According to the previous reports^51^, the cross-relaxation should dominate the above process among Er^3+^. Three possible cross-relaxation processes (^4^I_13/2_ + ^4^I_13/2_ → ^4^I_15/2_ + ^4^I_9/2_; ^4^I_13/2_ + ^4^I_11/2_ → I_15/2_ + ^4^F_9/2_; ^4^I_13/2_ + ^4^I_9/2_ → ^4^I_15/2_ + ^4^S_3/2_) are added in Fig. 2f.

As revealed in Fig. 2g, the energy levels of ^4^I_13/2_ and ^4^I_9/2_ play a crucial role in UC emissions for MLnS_2_:Er^3+^. To deeply understand the essence of highly efficient UC emissions for NaYS_2_:Er^3+^, the decay time curves of MLnS_2_:Er^3+^ were measured (Supplementary Figs. 14, 15) and the lifetimes histogram of ^4^I_13/2_ and ^4^I_9/2_ are performed as Fig. 2h, i, corresponding lifetimes constants are listed in Supplementary Table 3. It is very interesting that the decay time constants for α-MLnS_2_:Er^3+^ (2.4–2.84 ms for ^4^I_9/2_; 4.69–7.08 ms for ^4^I_13/2_) are significantly shorter than that of β-MLnS_2_:Er^3+^ (7.45-9.24 ms for ^4^I_9/2_; 13.12-30.27 ms for ^4^I_13/2_). M^+^ and Ln^3+^ are randomly disordered on an identical lattice site in α-MLnS_2_ with cubic structure, inevitably leading to the intensified negative energy exchange. While the Er^3+^ in β-MLnS_2_ are ordered separated by NaS6 octahedral layers along the c-axis direction, which blocks the cross-relaxation between each other to a certain extent. Therefore, in virtue of different cation distribution, the long decay time of the ^4^I_13/2_ level in the β compared to the α polymorph is observed. Excitingly, the NaYS_2_:Er^3+^ phosphors have the longest lifetimes in all the samples, reaching 30.27 ms and 9.24 ms for ^4^I_13/2_ → ^4^I_15/2_ and ^4^I_9/2_ → ^4^I_15/2_ transitions, respectively. As mentioned in Fig. 2f, the UC emissions origin in the electrons populating process via ESA from low to high energy levels step by step. Significantly, the delayed lifetimes in NaYS_2_:Er^3+^ mean that the electrons on ^4^I_13/2_ and ^4^I_9/2_ levels stay longer, which is in favor of being re-simulated to higher green energy levels (^4^S_3/2_/^2^H_11/2_), bringing about efficient green UC emissions. This is in line with the results in Fig. 2c, d, that show the stronger UC emissions and the longer lifetimes of ^4^I_13/2 _→ ^4^I_15/2_ and ^4^I_9/2_ → ^4^I_15/2_ transitions in MLnS_2_:Er^3+^ phosphors. From the UC emission spectra and luminescent decay curves as a function of temperature ranging from 10 - 300 K in Supplementary Figs. 16–21, the radiative and non-radiative rates of Er^3+^ in NaYS_2_ are significantly shorter than that of the other UC materials^52^. It can be concluded that the long lifetimes of NaYS_2_:Er^3+^ phosphors originate from the long radiative and non-radiative lifetimes of Er^3+^ (See Supplementary Fig. 2 and Supplementary Note 3). The long lifetimes are caused by the crystal structure and low phonon energy of NaYS_2_ (Supplementary Fig. 6) that lead to less non-radiative transition and weak electron-phonon coupling in NaYS_2_ host.

In order to understand qualitatively the UC luminescence mechanism in Er^3+^, a set of rate equations were established based on the UC process in MLnS_2_:Er^3+^ phosphors (in Fig. 2f, See Supplementary Note 4):

1 $${I}_{{green}}\propto \frac{{\sigma }_{13}^{2}{\sigma }_{35}{\rho }^{3}}{{C}_{8}\left({R}_{1}+{\delta }_{13}\rho \right)\left({R}_{3}+{{R}^{{\prime} }}_{32}+{\sigma }_{35}\rho \right)},$$

where *R*_*1*_ and *R*_*3*_ represent the radiative rates of ^4^I_13/2_ and ^4^I_9/2_ level of Er^3+^, and *R’*_*ij*_ is non-radiative rate from level *i* to level *j*. *ρ* is the laser photon number density. *σ*_*ij*_ denotes the absorption cross-section between level *i* and *j* of Er^3+^. *C*_*8*_ represents cross-relaxion process of ^4^I_13/2_+^4^I_9/2_→^4^I_15/2_+^4^S_3/2_. It can be concluded from Eq. (1) that the *I*_*green*_ value shows a cubic dependence on the laser photon number density, which means that the green emission results from a three-photon process, coinciding with the results in Supplementary Fig. 8. The green emission intensity is proportional to ${\sigma }_{35}$ and ${\sigma }_{13}^{2}$, and the larger absorption cross-section of ^4^I_9/2_ level, especially for ^4^I_11/2_ level can lead to the strong green emission. The *I*_*green*_ is inversely proportional to the *R*_*1*_, *R*_*3*_ and *R'*_*32*_, that is, it is proportional to the radiative lifetime (τ) of ^4^I_13/2_, ^4^I_9/2_ levels. From the UC spectra and dynamics at low temperature (Supplementary Figs. 16–21), the radiative lifetimes of Er^3+^ is much longer than that of commercial NaYF_4_:Yb^3+^, Er^3+^^52^, leading to efficient green UC emissions in NaYS_2_:Er^3+^. In addition, the long non-radiative lifetimes of Er^3+^ lead to weak *C*_*8*_ and induce strong green UC luminescence. Therefore, such efficient UC is dominated by the advantage of the exceptionally long lifetimes of the excited-state levels of Er^3+^.

**Fig. 3:**
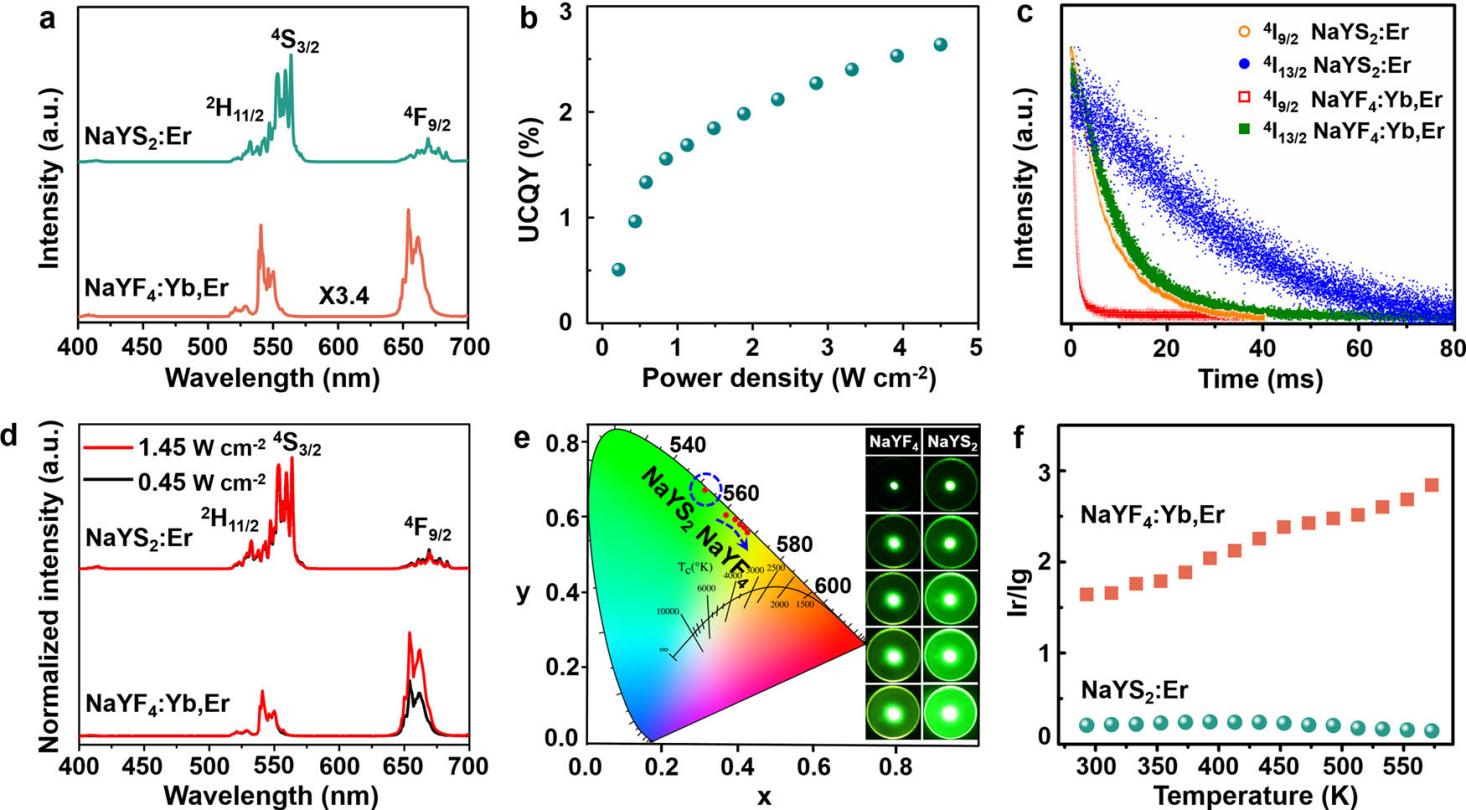
Luminescence properties comparison of NaYS_2_:Er^3+^ (1532 nm) and commercial NaYF_4_:Yb^3+^, Er^3+^ (980 nm). **a** Normalized UC luminescence spectra of NaYS_2_:Er^3+^ under 1532 nm excitation and NaYF_4_:Yb^3+^, Er^3+^ under 980 nm excitation with the same power density (0.45 W cm^−2^). **b** UCQYs of NaYS_2_:Er^3+^ under 1532 nm excitation at different power densities. **c** Decays curves of ^4^I_9/2_→^4^I_15/2_ and ^4^I_13/2_→^4^I_15/2_ transitions of Er^3+^ in NaYF_4_:Yb^3+^, Er^3+^ and NaYS_2_:Er^3+^. **d** Normalized UC luminescence spectra of NaYS_2_:Er^3+^ (1532 nm) and NaYF_4_:Yb^3+^, Er^3+^ (980 nm) at 0.45 and 1.45 W cm^−2^. **e** CIE chromaticity coordinates and luminescence detail images of NaYS_2_:Er^3+^ under 1532 nm excitation and NaYF_4_:Yb^3+^, Er^3+^ under 980 nm excitation as the power density increase from 0.45 to 1.45 W cm^−2^. **f** Temperature dependence of UC luminescence ratios between red and green emissions. Source data is provided in this paper.

Rare earth-doped β-NaYF_4_ (such as β-NaYF_4_:Yb^3+^, Er^3+^) has been recognized as the most efficient UC material. Therefore, the commercial NaYF_4_:Yb^3+^, Er^3+^ phosphor of similar size (Supplementary Fig. 22) is selected as a comparison with the NaYS_2_:Er^3+^ phosphor. Figure 3a shows the normalized UC luminescence spectra of NaYS_2_:Er^3+^ under 1532 nm excitation and NaYF_4_:Yb^3+^,Er^3+^ under 980 nm excitation with the same power density (0.45 W cm^-2^). It is amazing to observe that the green emission intensity of NaYS_2_:Er^3+^ is 3.4 times stronger than that of NaYF_4_:Yb^3+^, Er^3+^ (Fig. 3a). The ratio of green to red emissions is much higher in NaYS_2_:Er^3+^, suggesting the much weaker non-radiative transition from ^4^I_9/2_→^4^I_11/2_ and further confirming the weak electron-phonon coupling. Supplementary Figs. 23 demonstrates that NaYS_2_:Er^3+^ displays more efficient UC emission than NaYF_4_:Yb^3+^, Er^3+^ as increasing the illuminating power density. The luminescence brightness of NaYS_2_:Er^3+^ and NaYF_4_:Yb^3+^, Er^3+^ at different power densities are measured, as displayed in Supplementary Figs. 24, 25. The brightness of NaYS_2_:Er^3+^ is as high as 8224 cd/m^2^, while it is 3985 cd/m^2^ for NaYF_4_:Yb^3+^, Er^3+^ at the excitation power density of 1532 and 980 nm of 1.45 W cm^−2^. To maintain the same photon flux, the UC brightness of NaYS_2_:Er^3+^ excited with 1532 nm (0.27–0.95 W cm^−2^) and NaYF_4_:Yb^3+^, Er^3+^ excited with 980 nm (0.45-1.45 W cm^−2^) are compared, as shown in Supplementary Fig. 25 and Supplementary movie 1. It can be seen that the brightness of NaYS_2_:Er^3+^ is also higher than that of NaYF_4_:Yb^3+^, Er^3+^. The UCQYs of MLnS_2_:Er^3+^ (M = Li, Na, K; Ln = La, Y, Lu, Gd) are measured using the typical method with two detectors in an integrating sphere (See Supplementary Fig. 1 and Supplementary Note 1)^53^, under the illumination of 1532 nm as a function of excitation power density of 0-4.5 W cm^−2^, as displayed in Fig. 3b and Supplementary Fig. 26. The MLnS_2_:Er^3+^ show high UCQYs under 1532 nm excitation with the power density of 4.5 W cm^−2^ ranging of 0.5–2.6% (Supplementary Fig. 26 and Supplementary Data 1). The UCQY of NaYS_2_:Er^3+^ is best, reaching 2.6%, which is much higher than that of NaYF_4_:Yb^3+^, Er^3+^ (1.3%) under the illumination of 980 nm with the same power density (Supplementary Fig. 27). We estimated the theoretical UCQY of NaYS_2_:Er^3+^ under the weak excitation from the rate equations (See Supplementary Note 2) combining with the decay times at low temperature (10 K) and room temperature (Supplementary Table 4). The estimated green UCQY in NaYS_2_:Er^3+^ is 7.6% and 3.0% at low temperature and room temperature, respectively. Compared to the other representative UC materials, the UCQY of NaYS_2_:Er^3+^ significantly improves under around 1532 nm excitation, whereas the excitation power density in our system is much smaller than that of the literatures^53–56^. For example, the reported UCQYs of LiYF_4_:Er^3+^ and SrF_2_:5%Yb^3+^, 1%Er^3+^ are <0.2% with the power density of 150–850 W cm^−2^ excited at 1532 nm and 1490 nm, respectively, which are markedly higher than that of 4.5 W cm^−2^ ^57^. Furthermore, NaYS_2_:Er^3+^ show bright UC luminescence under 980 nm excitation. The UCQYs of NaYS_2_:Er^3+^ were recorded with varying power density at 980 nm (Supplementary Fig. 28). It was measured to be 0.4% with the power density of 4.5 W cm^−2^. Since the absorption cross-section of Er^3+^ at 980 nm is much smaller than that of at 1532 nm^58^, the UC intensity/efficiency of NaYS_2_:Er^3+^ excited at 980 is lower than that of 1532 nm excitation (Supplementary Fig. 29). The UC emission intensity of NaYS_2_:Er^3+^ compared with commercial NaYF_4_:Yb^3+^, Er^3+^ is shown in Supplementary Fig. 30. The UC emission intensity of NaYS_2_:Er^3+^ is just lower 8.1 fold than that of NaYF_4_:Yb^3+^, Er^3+^ excited at the same power density of 980 nm (0.45 W cm^−2^; Supplementary Fig. 30). Considering the higher concentration (18%) and absorption cross-section (1.2 × 10^−20^ cm^2^) at 980 nm of Yb^3+^ in NaYF_4_:Yb^3+^, Er^3+^ than that of Er^3+^ (5%, 1.7 × 10^−21^ cm^2^), NaYS_2_:Er^3+^ is indeed efficient and a promising UC material for 980 nm pumping. It should be noted that the NaYS_2_:Er^3+^-based ESA process not only obtains the more efficient UC emissions, but also expands its pumping wavelength to NIR II region. It should be highlighted that green and red UC emissions in NaYS_2_:Er^3+^ are from a three-photon process under 1532 nm excitation, while they are a two-photon process in the NaYF_4_:Yb^3+^, Er^3+^ phosphor under 980 nm excitation. It is difficult for the former one to obtain highly efficient UC. In fact, the multi-photons UC is also possible to be more effective than two-photon UC, owing to the enriched energy levels and diversity of transitions in UC^59^. Such efficient UC emissions-induced ESA may be ascribed to the significantly longer lifetimes of ^4^I_9/2_ and ^4^I_13/2_ levels of Er^3+^ in NaYS_2_:Er^3+^, which are 9.4 fold and 3.05 fold longer than that of NaYF_4_:Yb^3+^, Er^3+^ (0.98 ms and 9.92 ms for ^4^I_9/2_ and ^4^I_13/2_ levels, as shown in Fig. 3c and Supplementary Table 3). UC emission as a multistep process not only relies on the absorption cross-section of rare earth ions and excitation light intensity, but also strongly depends on the electrons population on the intermediate energy levels. The long lifetime of the intermediate energy levels indicates more electrons resting on them for longer time, is of significance to realize efficient UC in NaYS_2_:Er^3+^, in accordance to the previous reports^60^. Based on the theory of multi-phonon relaxation (See Supplementary Note 3, Supplementary Fig. 21, and Supplementary Table 5), both the radiative and non-radiative rates in NaYS_2_:Er^3+^ are much smaller than that of NaYF_4_:Yb^3+^, Er^3+^ ^52^. These can be explained by the ordered layered structures and low phonon energy of NaYS_2_ host. Such long lifetimes of rare earth ions in MLnS_2_ hosts are in favor of multi-photon UC, providing novel approach to develop efficient UC materials.

In addition, the UC spectra of NaYS_2_:Er^3+^ remain unchanged with varying the excitation power density under 1532 nm excitation (Fig. 3d). The CIE chromaticity coordinates and digital camera images in Fig. 3e demonstrate that the emission color of NaYF_4_:Yb^3+^, Er^3+^ gradually shifts from green (0.3671, 0.6057) to red (0.4290, 0.5596) region, while NaYS_2_:Er^3+^ shows higher brightness and excellent color stability with concentrating in the green emission region (0.3131, 0.6737) by varying the pumping power. In addition, the UC luminescence ratio of red to green emissions in NaYF_4_:Yb^3+^, Er^3+^ significantly increases, whereas it changes little for NaYS_2_:Er^3+^ (Fig. 3f). These advantages can be attributed to the lower non-radiation in NaYS_2_:Er^3+^. These indicate that NaYS_2_:Er^3+^ phosphors have high stability towards light and temperature.

### NIR photodetection and underwater optical communication applications

**Fig. 4:**
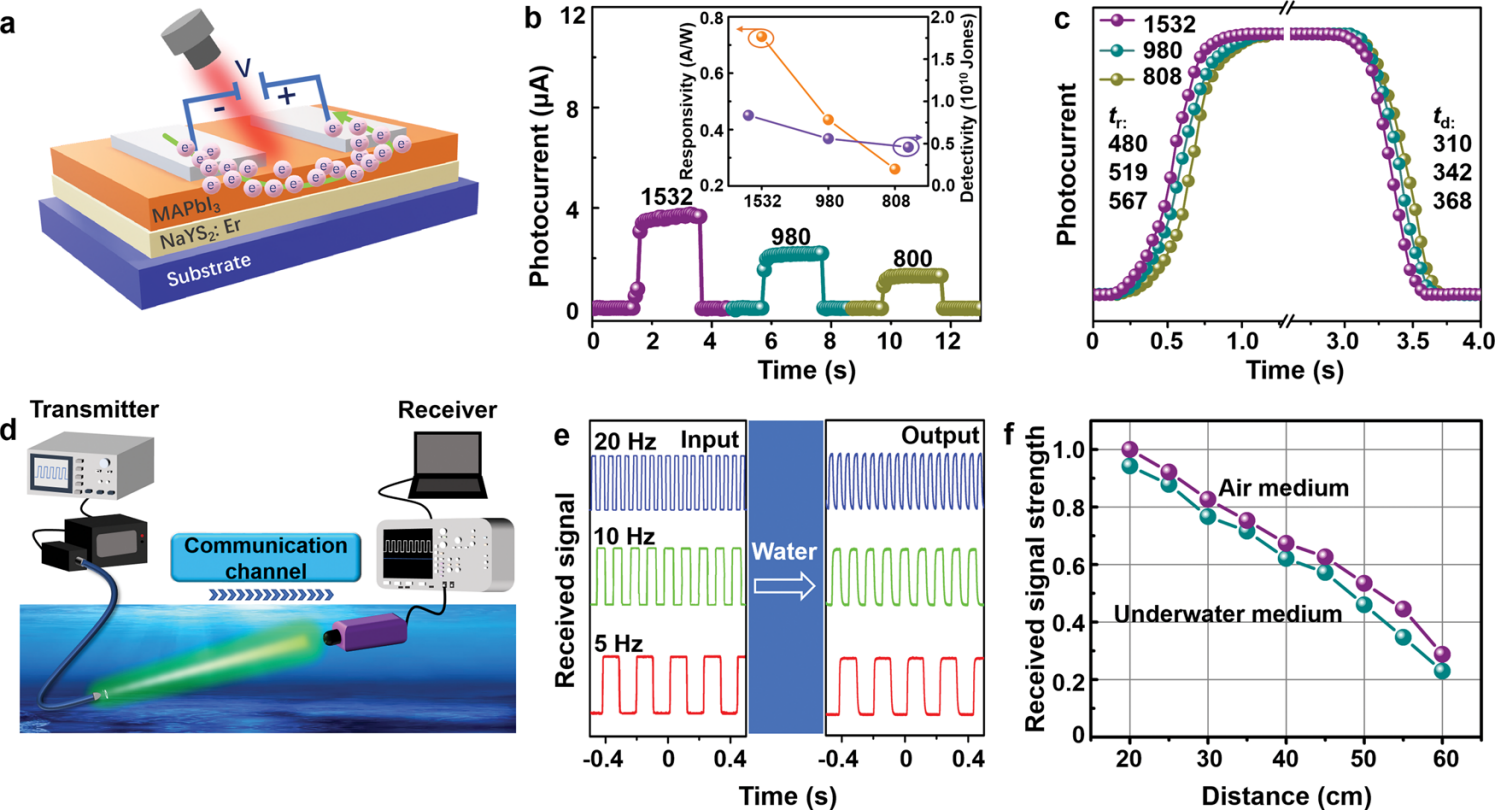
Multispectral narrowband NIR photodetection and underwater information transmission of NaYS_2_:Er^3+^ phosphor. **a** Schematic illustration of structure and working mechanism in the NaYS_2_:Er^3+^/MAPbI_3_ PDs. **b** Photocurrent response of NaYS_2_:Er^3+^/MAPbI_3_ PDs under 808, 980, and 1532 nm excitation at power density of 5 mW cm^−2^, respectively (inset: the responsivity and detectivity of NaYS_2_:Er^3+^/MAPbI_3_ device under separately 808, 980, and 1532 nm excitation). **c** On-off switching currents at lowest detectable excitation power density of NaYS_2_:Er^3+^/MAPbI_3_ device under 808, 980, and 1532 nm, respectively. **d** Schematic illustration of the NaYS_2_:Er^3+^ underwater information transmission in seawater. **e** Input and output signal information of transmitter and receiver at different frequencies (Hz) with the seawater depth of 0.6 m. **f** Received signal strength of NaYS_2_:Er^3+^ in air and water at different distance between transmitter and receiver. Source data is provided in this paper.

Narrowband NIR photodetection has been attracting substantial attention in diverse areas, including biological analysis, bio-imaging/sensing, and encrypted communications etc^61,62^. As a proof of concept, we designed and fabricated narrowband responsive NIR photodetectors (PDs) using a simple NaYS_2_:Er^3+^/MAPbI_3_ hybrid. As illustrated in Fig. 4a, a high-quality MAPbI_3_ film acting as the photon-to-current material was spin-coated on the top of a NaYS_2_:Er^3+^ film (the top view SEM image of the NaYS_2_:Er^3+^/MAPbI_3_ hybrids is shown in Supplementary Fig. 31), Then the silver electrodes were deposited on the MAPbI_3_ film. The working mechanism of the NaYS_2_:Er^3+^/MAPbI_3_ PD can be explained in Supplementary Fig. 32. Briefly, the NaYS_2_:Er^3+^ phosphor can absorb the incident NIR photons peaking around 808, 980, and 1532 nm, respectively, and convert them to visible light in the spectral range of 400–700 nm through photon UC. The up-converted light can be efficiently absorbed by the perovskite MAPbI_3_ with a narrow band gap (~800 nm), thereby producing photocurrents. Figure 4b displays the typical on-off photocurrent-time (*I-t*) response curves of the NaYS_2_:Er^3+^/MAPbI_3_ device separately under 808, 980, and 1532 nm illumination with an incident light intensity of 5 mW cm^−2^ at the bias voltage of 1 V. The photocurrents are 1.26, 2.19, and 3.78 µA in NaYS_2_:Er^3+^/MAPbI_3_ PDs for 808, 980, and 1532 nm. This is in well agreement with the UC luminescence intensities for the NaYS_2_:Er^3+^ phosphor under corresponding excitations, respectively, and shown in Supplementary Fig. 32. Three representative parameters (See Supplementary Note 5) can be used to characterize the performance of the PDs, namely photo-responsivity (*R*), detectivity (D*) and external quantum efficiency (*EQE*). As exhibited in the insets of Fig. 4b and Supplementary Fig. 33, R, D* and *EQE* of the NaYS_2_:Er^3+^/MAPbI_3_ PDs are determined to be 0.26 A/W, 0.44 A/W, and 0.73 A/W; 0.46$\times$10^10^ Jones, 0.56$\times$10^10^ Jones, and 0.84$\times$10^10^ Jones; 39%, 55%, and 59% for the 808, 980, and 1532 nm light, respectively. For the photodetector, EQE is equal to the number of electron-hole pairs per second collected to produce the photocurrent*I*_ph_, divided by the number of incident photons per second. In a detector with 100% EQE, R = 1 A/W for photon energy *E*_ph_ = 1 eV^63^. The photoconductive gain leads to the EQE > 50% when applied a bias in PDs, similar to previous reports^64–66^. In general, UC visible emissions of NaYS_2_:Er^3+^ under 1532 nm excitation excite the semiconductor (MAPbI_3_) to produce electron-hole (e-h) pairs. Since holes generally move more slowly than electrons in semiconductors, N electrons have already been collected by the electrode when the slower-moving hole is searched by the electrode under an applied electric field. The device at 1532 nm shows the best performance owing to the most effective UC at this excitation wavelength. The Fig. 4c shows that the photon-response times, which were extracted from the dynamic response curves of photocurrents of the device under 808, 980, or 1532 nm light illumination. The NaYS_2_:Er^3+^/MAPbI_3_ PD exhibits response times in the range of 310–570 ms. As shown in Supplementary Fig. 34, the photodetection thresholds for the NaYS_2_:Er^3+^/MAPbI_3_ PDs reached below 2 mW cm^−2^ for 808 and 980 nm light, particularly below 1 mW cm^−2^ for 1532 nm light. Compared to other representative NIR PDs, our PDs based on NaYS_2_:Er^3+^ phosphors demonstrate the excellent capability for multi-wavelength detection and good performance (Supplementary Table 6).

Visible light communication is environmentally friendly, and can realize low energy consumption communication, effectively avoid the weaknesses of radio communication, such as electromagnetic signal leakage, and will also interact and integrate with WiFi, cellular network technologies, bringing innovative applications in the fields of Internet of Things, navigation, and high-speed rail etc^67^. For example, the development and collection of marine resources are closely related to underwater optical communication. In contrast to high absorption of the UV and NIR wavelength, the low absorption at the visible wavelength (green light) of seawater is suitable for under seawater communication, which usually requires visible light source with high power for long-distance seawater transmission^68^. As known, optical fiber communication based on 1532 nm is mature technology, which can be easily coupled into optical fiber with low coupling and transmission losses. Based on the above results, NaYS_2_:Er^3+^ phosphors can efficiently convert 1532 nm to green emissions, which simultaneously combines the advantages of NIR optical fiber communication and visible optical communication. As a proof of concept demonstration, the NIR-II responsive NaYS_2_:Er^3+^ phosphors are used to construct the UC underwater information transmission system, as illustrated in Fig. 4d. The system is composed of three parts: transmitter, channel, and receiver. Firstly, the NaYS_2_:Er^3+^ phosphors mixed with epoxy resin were packaged on the optical fiber, and 1532 nm laser diode (LD) was coupled into the optical fiber which further simulated the NaYS_2_:Er^3+^ phosphors, then generating UC green emission lights. The input signal of 1532 nm with different frequencies was adjusted by the signal generator, Then, the emitted UC lights carrying information passed through the seawater, and collected by receivers. As shown in Fig. 4e, the input signals are the pulsed 1532 nm lights at different working frequency, and the output signals present the 564 nm green emissions of NaYS_2_:Er^3+^ excited by the 1532 nm. The green UC emission lights remain the frequency after passing seawater over a distance of 0.6 m (See Supplementary Movie 2). In addition, the received signal strengths of UC green emissions are compared in air and seawater medium at the fixed distance of 1 m. It is clearly observed from Fig. 4f that the reduced rates of green emission of NaYS_2_:Er^3+^ are basically the same in these two media. These suggest that such NIR II-responsive NaYS_2_:Er^3+^ phosphors can effectively convert 1532 nm light into green light to realize underwater optical communication. Because of the large loss of visible light in optical fiber, the transmission distance of visible light cannot be matched with that of 1532 nm light in an optical fiber. Therefore, the visible light communication based on the UC green emission of NaYS_2_:Er^3+^ phosphor using 1532 nm as light source possesses great value in underwater optical communication.

## Discussion

In summary, the Er^3+^ doped MLnS_2_ (M = Li, Na, K; Ln = La, Y, Gd, Lu; phases: cubic, or trigonal) phosphors were successfully prepared through the gas-solid reaction method. All of them demonstrate the efficient UC emissions under the illumination of NIR II 1532 nm based on the ESA process. By comparison, β-NaYS_2_ phosphor is recognized as the most UC material in MLnS_2_:Er^3+^ under 1532 nm excitation. It can be explained by ordered layer structure of β phase instead of disordered structure of α phase, as well as exceptionally long lifetimes of excited state levels of NaYS_2_:Er^3+^. β-NaYS_2_:Er^3+^ displays remarkably higher UCQY and brightness, and much better spectral stability of lights illumination and temperature than those of commercial NaYF_4_:Yb^3+^, Er^3+^ phosphor. β-NaYS_2_:Er^3+^ realizes a breakthrough UC efficiency as high as 2.6% under 1532 nm excitation, and the brightness of NaYS_2_:Er^3+^ is 8224 cd/m^2^ under 1532 nm irradiation. Such high optical performances can be assigned to low non-radiative transition and electron-phonon coupling in NaYS_2_:Er^3+^ phosphor. Furthermore, we designed and fabricated the sensitive narrowband responsive NIR PDs at wavelength of 808, 980, and 1532 nm, and the UC green light underwater communication application. Our work provides a strategy for constructing NIR II responsive UC materials and expanding the scope of applications.

## Methods

### Materials

Lanthanide oxides (Y_2_O_3_, Lu_2_O_3_, La_2_O_3_, Gd_2_O_3_, or Er_2_O_3_; 99.99%), and alkaline metal carbonates (Na_2_CO_3_, K_2_CO_3_, or Li_2_CO_3;_ A.R.) were used as the primary materials. Carbon disulfide (CS_2_, Aladdin, ≥99%) was employed as the sulfur source.

### Synthesis of micron-sized MLnS_2_:Er^3+^ phosphors

MLnS_2_:Er^3+^ (M = Li, Na, or K; Ln = Y, Lu, La, or Gd) phosphors were fabricated by the gas-solid reaction method. In the typical synthesis process, the Ln_2_O_3_ (15 mmol) and M_2_CO_3_ (16.5 mmol) were mixed and thoroughly ground in an agate mortar for 30 min according to the 1:1.1 ratio, and the doping concentrations of Er^3+^ were 2, 5, 10, and 15 mol%, respectively. The mixture was transferred to the quartz boat and placed into the tube furnace. The samples were heated to 773 K under argon (Ar) atmosphere, and then saturated vapor of CS_2_ (40 kPa) was flowed in Ar at a flow rate of 50 mL/min. The as-prepared samples were obtained after calcining the mixture at 1073, 1173, 1273, or 1373 K for two hours and washing by water and ethanol for several times.

### Structure and morphology characterization

The X-ray diffraction (XRD) patterns of samples were characterized by the Shimadzu XRD-6000 X-ray diffractometer with a Cu Kα (λ = 0.1541 nm). Scanning electron microscopic (SEM) images of samples were performed by the Hitachi S-4800 scanning electron microscope. Transmission electron microscopic (TEM) and high-resolution transmission electron microscopic (HRTEM) images of samples were measured by using a JEM-2100F transmission electron microscope.

### Optical properties measurement

All samples were pressed into the plate shape with the thickness of 2 mm for optical characterization. The absorption spectra of the samples were recorded on a Lambda 750 UV–Vis-NIR spectrophotometer (Perkin-Elmer, USA). The Raman spectra were measured by the Renishaw InVia Raman Microscope (maximum power: 150 W) equipped 532 nm laser as a source of excitation, and the absolute accuracy is 0.55 cm^−1^ for λ = 532 nm. The UC luminescence spectra and decay curves were measured by a Jobin Yvon iHR550 monochromator equipped with R928 and H10220B-75 photomultiplier tubes from Hamamatsu Photonics using the light sources of laser diodes (808, 980, and 1532 nm; maximum power of 2.0 W), and a pulsed work Horizon OPO laser, for signal collection from 400 nm to 1700 nm with a step length of 0.2 nm. The decay profiles were collected using a Tektronix DPO 5104 digital oscilloscope. A closed-cycle helium gas cryostat (Advanced Research Systems, Model CSW-202) equipped with a Lakeshore Model 331 temperature controller was used to control the sample temperature in the 10–300 K range. The underwater optical communication test was performed by a self-designed system, the signal transmitter was composed of a signal generator and 1532 nm LD, Jobin Yvon iHR550 monochromator and oscilloscope constitute the signal receiver. The UCQYs of MLnS_2_:Er^3+^ were directly measured using a commercial setup (XPQY-EQE-SolTM 1.7, Guangzhou Xi Pu Optoelectronics Technology Co., Ltd.) equipped with an integrated sphere (GPS-4P-SL, Labsphere).

## Supplementary information

Supplementary Information

Description of Additional Supplementary Files

Supplementary Data 1

Movie1

Movie2

## Data Availability

All data generated or analysed during this study are included in this published article and its supplementary information files. Source data are provided with this paper.

## Source data

Source Data
