# Publisher Correction: Towards highly efficient NIR II response up-conversion phosphor enabled by long lifetimes of Er^3+^

**DOI:** 10.1038/s41467-022-34690-y

**Published:** 2022-11-17

**Authors:** Xiumei Yin, Wen Xu, Ge Zhu, Yanan Ji, Qi Xiao, Xinyao Dong, Ming He, Baosheng Cao, Na Zhou, Xixian Luo, Lin Guo, Bin Dong

**Affiliations:** 1 School of Physics and Materials Engineering, Dalian Minzu University, 18 Liaohe West Road, Dalian, 116600 China; 2 School of Chemistry and Environment, Beijing University of Aeronautics & Astronautics, 37 Xueyuan Road, Beijing, 100191 China

**Keywords:** Optical materials and structures, Materials for optics

Correction to: *Nature Communications* 10.1038/s41467-022-34350-1, published online 01 November 2022

The original version of this Article contained an error in the spelling of the author Bin Dong, which was incorrectly given as Dong Bin. This has now been corrected in both the PDF and HTML versions of the Article.

