# Supplementary material for: Towards highly efficient NIR II response up-conversion phosphor enabled by long lifetimes of Er3+
Source: Nat Commun. 2022 Nov 1;13:6549. doi: 10.1038/s41467-022-34350-1 (PMC9626601; doi:10.1038/s41467-022-34350-1)
Supplement: Supplementary file 1 — Supplementary Information [file 41467_2022_34350_MOESM1_ESM.pdf]

Supplementary Information

Towards highly efficient NIR II response up-conversion phosphor enabled by long lifetimes of Er^{3+}

Xiumei Yin[1†], Wen Xu[1,2†], Ge Zhu[1], Yanan Ji[1], Qi Xiao[1], Xinyao Dong[1], Ming He[1], Baosheng Cao[1], Na Zhou[1], Xixian Luo[1]*, Lin Guo [2]*, Bin Dong [1]*

[1] School of Physics and Materials Engineering, Dalian Minzu University, 18 Liaohe West Road, Dalian 116600, P. R. China.

[2]School of Chemistry and Environment, Beijing University of Aeronautics & Astronautics, 37 Xueyuan Road, Beijing 100191, PR China.

[†]These authors contributed equally to this work.

Corresponding authors: Prof. Bin Dong (dong@dlnu.edu.cn), Prof. Xixian Luo (luoxx@dlmu.edu.cn); Prof. Lin Guo (guolin@buaa.edu.cn).

I. Supplementary Methods

Supplementary Note 1 - Measurement of UCQYs

The UCQYs of $MLnS_2:Er^{3+}$ were directly measured using a commercial setup (XPQY-EQE-SolTM 1.7, Guangzhou Xi Pu Optoelectronics Technology Co., Ltd.) equipped with an integrated sphere (GPS-4P-SL, Labsphere), which was first developed by Prof. Frank C. J. M. van Veggel in 2010 (Supplementary Ref. 1: Nanoscale, 2010, 2, 1417-1419), as displayed in Supplementary Figure 1.

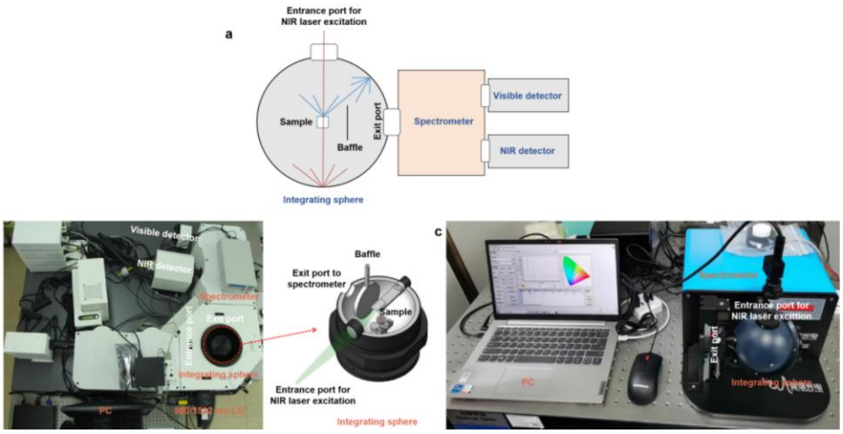

Supplementary Figure 1. Experimental setup for UCQYs measurement. (a) Schematic diagram and photos of the experimental setup to determine the UCQY under near infrared (NIR) laser excitation via an integrating sphere: (b-c) Actual measurement setup for UCQY based on FLS-1000 from Edinburgh Instruments and XPQY-EQE-SolTM 1.7 from Guangzhou Xi Pu Optoelectronics Technology (The detailed internal structure of integrating sphere is shown in (b) on the right).

Firstly, the samples of micro-sized $MLnS_2:Er^{3+}$ or $NaYF_4:Yb^{3+},Er^{3+}$ (~500 mg) were encapsulated in barium sulfate cells with a thickness of 2 mm, which were located in the integrating sphere. Then, the samples were directly excited with illumination area of 0.28 cm^2 by near infrared (NIR) laser diodes. The 1532 nm (MW-GX-1532/2000mW, Changchun Laser Optoelectronics Technology Co., Ltd.) and 980 nm lasers (MW-GX-980/2000mW, Changchun Laser Optoelectronics Technology Co., Ltd.) were employed to pump $MLnS_2:Er^{3+}$ and/or $NaYF_4:Yb^{3+},Er^{3+}$, respectively. The photons number of the pumping laser, the remained photons number of the pumping laser after pumping the samples, and the photons number of UC emissions were collected by the spectrometer equipped with two detectors of Si-CCD and InGaAs, respectively. The Si-CCD detector (S7031-1006, Hamamatsu Photonics) with the wavelength

response range of 200-1100 nm was used to record the UC emissions signals from samples. The InGaAs detector (G9204-512, Hamamatsu Photonics) with the wavelength response range of 800-1670 nm was adopted to collect the excitation light signals. Before the measurement of UCQY, the response of the such system in photon flux, including the spectral response of the integrating sphere, spectrometer, and two detectors was carefully calibrated using HL-3 Radiometric calibrated light source (Ocean Optics). The signal of NIR excitation detected by InGaAs detector should be multiplied by a factor (~50).

For the measurement, the photons number of the pumping laser were directly recorded by placing a blank sample cell in the integrated sphere. After that, we put the sample cell loading with $MLnS_2:Er^{3+}$ or $NaYF_4:Yb^{3+},Er^{3+}$ phosphors into the integrating sphere, and further measured the photons number of remaining laser after absorbing by $MLnS_2:Er^{3+}$ or $NaYF_4:Yb^{3+},Er^{3+}$ phosphors, and the UC emission photons number of $MLnS_2:Er^{3+}$ or $NaYF_4:Yb^{3+},Er^{3+}$ phosphors. The power density of pumping laser is 0-4.5 W cm^{-2}. Finally, the UCQYs of $MLnS_2:Er^{3+}$ phosphors as a function of power density were calculated through the ratio of the emission photons number of $MLnS_2:Er^{3+}$ or $NaYF_4:Yb^{3+},Er^{3+}$ phosphors to the photons number of laser absorbed by $MLnS_2:Er^{3+}$ or $NaYF_4:Yb^{3+},Er^{3+}$ phosphors. The measured emission range for UCQYs of all the samples are 400-750 nm.

It should be noted that, to ensure the accuracy of UCQYs, we also performed the UCQYs of $MLnS_2:Er^{3+}$ using Edinburgh Instruments (FLS-1000). In addition, we further verified the reliability of acquired UCQYs from both instruments by using the well-recognized $NaYF_4:Yb^{3+}$, Er^{3+} phosphors as a reference sample.

Supplementary Note 2 - Calculated UCQY from the rate equations

We propose the rate equations to calculate the UCQY of green emission, because the green UC emission is dominate in $NaYS_2:Er^{3+}$. When we consider the UC population process of green emission of Er^{3+} excited at 1532 nm under weak excitation, the intermediate energy levels of $^4I_{13/2}$ and $^4I_{9/2}$ play a crucial role in the UC emission process (Fig. 2f). To qualitatively analyse the UCQY in $NaYS_2:Er^{3+}$, a set of rate equations was established as follows:

$$\frac{dN_1}{dt} = \sigma_{01}\rho N_0 - R_1N_1 - R'_1N_1 \quad (1)$$

$$\frac{dN_3}{dt} = \sigma_{13}\rho N_1 - \sigma_{35}\rho N_3 - R_3N_3 - R'_3N_3 \quad (2)$$

$$\frac{dN_5}{dt} = \sigma_{35}\rho N_3 - R_5 N_5 - R'_5 N_5 \quad (3)$$

where N_0, N_1, N_3, and N_5 denote the electrons population densities of $^4I_{15/2}$, $^4I_{13/2}$, $^4I_{9/2}$, and ($^2H_{11/2}$+$^4S_{3/2}$) levels of Er^{3+}, respectively. R_1, R_3, and R_5 are the radiative rates of $^4I_{13/2}$, $^4I_{9/2}$, and ($^2H_{11/2}$+$^4S_{3/2}$) levels of Er^{3+}, respectively. R'_1, R'_2, R'_5 is non-radiative rate from $^4I_{13/2}$, $^4I_{9/2}$, and ($^2H_{11/2}$+$^4S_{3/2}$) levels of Er^{3+} to other energy levels. ρ is the laser photon number density. σ_{ij} denotes the absorption cross-section between level i and j of Er^{3+}.

Under weak excitation (μJ, 1532 nm), the decay time in the excited levels after the pump switches off (10 ns) can be described as:

$$\tau_{^4I_{13/2}-1532nm} = \frac{1}{\sigma_{13}\rho + R_1 + R_1^{'}} \ , \quad (4)$$

$$\tau_{^4I_{9/2}-1532nm} = \frac{1}{\sigma_{35}\rho + R_3 + R_3^{'}} \ . \quad (5)$$

The 980 nm photons cannot directly pump the $^4I_{13/2}$ and $^4I_{9/2}$ energy levels for green UC emission, thus the decay time of $^4I_{13/2}$ and $^4I_{9/2}$ energy levels under 980 nm excitation can be presented in simplified form as:

$$\tau_{^4I_{13/2}-980nm} = \frac{1}{R_1 + R_1^{'}}, \quad (6)$$

$$\tau_{^4I_{9/2}-980nm} = \frac{1}{R_3 + R_3^{'}}. \quad (7)$$

We further measured the decay times of $^4I_{13/2}$, $^4I_{9/2}$, and $^4S_{3/2}$ in $NaYS_2:Er^{3+}$ excited at 1532 nm and 980 nm at low temperance (10 K) and room temperature, as listed in Supplementary Table 4. The green UC in Er^{3+} is realized by the upward electron population of the intermediate states of $^4I_{13/2}$ and $^4I_{9/2}$ under 1532 nm excitation, while it is low related with these two energy levels for green UC emission under 980 nm excitation. Hence, the shortened lifetimes of $^4I_{13/2}$ and $^4I_{9/2}$ in $NaYS_2:Er^{3+}$ under 1532 nm excitation compared with the excitation of 980 nm can be induced by the upward electron population. Considering the three-photons UC for green emission, the UCQY in $NaYS_2:Er^{3+}$ can be estimated using the following equation:

$$UCQY = \frac{N_{emit}}{N_{abs}} = \frac{\rho_{^4I_{13/2}-up} \times \rho_{^4I_{9/2}-up} \times \rho_{^4S_{3/2}-down}}{3}, \quad (8)$$

where N_{abs} and N_{emit} is the absorption photons for excitation light and emission photons from samples, $\rho_{^4I_{13/2}-up}$ and $\rho_{^4I_{9/2}-up}$ are the probability of photons jumping to upper levels of $^4I_{9/2}$

and $^4S_{3/2}$, and $\rho_{^4S_{3/2}-\mathrm{down}}$ presents the probability of the emission photons. $\rho_{^4I_{13/2}-\mathrm{up}}$, $\rho_{^4I_{9/2}-\mathrm{up}}$, and $\rho_{^4S_{3/2}-\mathrm{down}}$ can be expressed as:

$$\rho_{^4I_{13/2}-\mathrm{up}} = \frac{\sigma_{13}\rho}{\sigma_{13}\rho + R_1 + R_1^{'}}, \tag{9}$$

$$\rho_{^4I_{9/2}-\mathrm{up}} = \frac{\sigma_{35}\rho}{\sigma_{35}\rho + R_3 + R_3^{'}}, \tag{10}$$

$$\rho_{^4S_{3/2}-\mathrm{down}} = \frac{R_5}{R_5 + R_5^{'}}, \tag{11}$$

in which they are determined to be 53%, 43%, and 100 % at low temperature; and 57.8 %, 28.9 %, and 53.3 % at room temperature, respectively. The estimated green UCQY in $NaYS_2:Er^{3+}$ is 7.6 % and 3.0 % at low temperature (10 K) and room temperature, respectively.

Supplementary Note 3 - UC dynamics as a function of temperature

In order to understand the temperature-dependent luminescent dynamics, the decay time constants as a function of temperature were characterized [Supplementary Ref. 2]. In the measurement of UC dynamics, a tunable Nd:YAG laser pumped OPO laser with the output wavelength of 192-2750 nm as the excitation source, with a pulse duration of 10 ns, repetition frequency of 10 Hz and line width of 4-7 cm^{-1}. Thereafter, the decay profiles were collected using a Tektronix DPO 5104 digital oscilloscope as shown in Supplementary Figure 2.

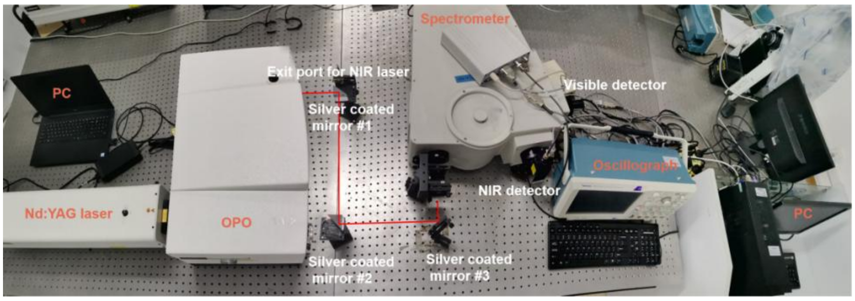

Supplementary Figure 2. Experimental setup for lifetime measurement. Actual experimental setup for photoluminescent lifetimes measurement consisting of Nd:YAG-OPO laser, spectrometer, and oscilloscope.

The radiative decay rate for an emission level rarely changes with temperature, while the nonradiative decay rate varies significantly with temperature, thus the luminescent lifetime τ can be expressed as:

$$\tau = \frac{1}{W + W(0)[1 - \exp(-\hbar\omega/kT)]^{-\Delta E/\hbar\omega}} \tag{12}$$

where W represents the radiative transition rate for the emission levels, $W(0)$ represents the nonradiative transition rate from the emission level to the nearest down level at 0 K, $\hbar\omega$ is the phonon energy for multi-phonon relaxation process and ΔE is the energy gap between the emission level and the nearest down level. The majority of experimental data can be well fitted by equation (12). W and $W(0)$ for $^4S_{3/2}$, $^4F_{9/2}$, $^4I_{11/2}$ levels were obtained, and the optimum fitting values were listed in Supplementary Table 5.

The UC emission spectra and luminescent decay curves of $NaYS_2:Er^{3+}$ phosphors under 1532 nm excitation as a function of temperature ranging of 10-300 K were recorded, as shown in Supplementary Figure 16-21. As UC emission intensities of Er^{3+} first increase with increasing the temperature from 10 K to 150 K, and then decreases with further increase of temperature for all the transitions ($^2H_{11/2}/^4S_{3/2}\rightarrow{}^4I_{15/2}$, $^4F_{9/2}\rightarrow{}^4I_{15/2}$ and $^4I_{9/2}\rightarrow{}^4I_{15/2}$), as shown in Supplementary Figure 17. With the initial increase of temperature, the relative population on the ground energy levels of the Er^{3+} will increase, leading to the increase of the excitation of Er^{3+}, as a consequence, the UC emission intensity increases. Meanwhile, the nonradiative relaxation processes will increase with the increase of temperature, leading to the quenching of UC emission. These two competing processes lead the appearance of an optimum of UC intensity at a certain temperature. Similar results were observed in $NaYF_4:Yb^{3+},Er^{3+}$ phosphors (Supplementary Ref. 2, Dalton Trans., 2014, 43, 6139–6147). Supplementary Figure 18-20 show the UC emission decay curves of $^4S_{3/2}\rightarrow{}^4I_{15/2}$, $^4F_{9/2}\rightarrow{}^4I_{15/2}$ and $^4I_{9/2}\rightarrow{}^4I_{15/2}$ transitions of Er^{3+} in $NaYS_2:Er^{3+}$, which decrease from 6.32 ms to 3.37 ms, 8.28 ms to 5.78 ms, and 22.55 ms to 9.24 ms with increasing the temperature from 10 K to 300 K, respectively. These shorted lifetimes of the above transitions of Er^{3+} in $NaYS_2:Er^{3+}$ can be fitted by the theory of multi-phonon relaxation (Supplementary Figure 21). As displayed in Supplementary Table 5, the radiative and non-radiative rates of $^4S_{3/2}$, $^4F_{9/2}$ and $^4I_{9/2}$ levels are determined to be 0.087 ms^{-1} and 0.011 ms^{-1}, 0.064 ms^{-1} and 0.014 ms^{-1}, and 0.025 ms^{-1} and 0.006 ms^{-1}, respectively. The radiative rates of Er^{3+} in $NaYS_2:Er^{3+}$ are significantly shorter (almost one order) than that of $NaYF_4:Yb^{3+},Er^{3+}$ (1.01 ms^{-1} and 1.24 ms^{-1} for $^4S_{3/2}$ and $^4F_{9/2}$; Supplementary Ref. 2, Dalton Trans., 2014, 43, 6139–6147). In addition, the non-radiative processes are also observed in $NaYS_2:Er^{3+}$, in which the non-radiative rates are much smaller than that of $NaYF_4:Yb^{3+},Er^{3+}$ (0.08 ms^{-1} and 0.90 ms^{-1} for $^4S_{3/2}$ and $^4F_{9/2}$; Supplementary Ref. 2, Dalton Trans., 2014, 43, 6139–6147), owing to the smaller phonon energy of $NaYS_2:Er^{3+}$. From the above results,

we can conclude that the Er^{3+} excited states in $NaYS_2$:Er^{3+} are with exceptionally long lifetimes, contributed by both the long radiative and non-radiative component.

Supplementary Note 4 - Transition rate equation for green emission in $MnLnS_2$:Er^{3+}

In order to understand qualitatively the UC luminescence mechanism in Er^{3+}, a set of rate equations were established based on the well-known UC process:

$$\frac{dN_1}{dt} = R'_{21}N_2 + \sigma_{01}\rho N_0 - \sigma_{13}\rho N_1 - C_6N_1^2 - C_7N_1N_2 - C_8N_1N_3 - R_1N_1 \quad (13)$$

$$\frac{dN_2}{dt} = R'_{32}N_3 - \sigma_{24}\rho N_2 - R'_{21}N_2 - C_7N_1N_2 - R_2N_2 \quad (14)$$

$$\frac{dN_3}{dt} = R'_{43}N_3 + \sigma_{13}\rho N_1 + C_6N_1^2 - \sigma_{35}\rho N_3 - R'_{32}N_3 - C_8N_1N_3 - R_3N_3 \quad (15)$$

$$\frac{dN_4}{dt} = R'_{54}N_5 + \sigma_{24}\rho N_2 + C_7N_1N_2 - R'_{43}N_4 - R_4N_4 \quad (16)$$

$$\frac{dN_5}{dt} = \sigma_{35}\rho N_3 + C_8N_3 - R'_{54}N_5 - R_5N_5 \quad (17)$$

$$N_{Er} = N_0 + N_1 + N_2 + N_3 + N_4 + N_5 \quad (18)$$

where N_0, N_1, N_2, N_3, N_4, and N_5 denote the population densities of $^4I_{15/2}$, $^4I_{13/2}$, $^4I_{11/2}$, $^4I_{9/2}$, $^4F_{9/2}$, and ($^2H_{11/2}$+$^4S_{3/2}$) levels of Er^{3+}, respectively. N_{Er} is the nominal ion density corresponding to Er^{3+} doping concentration. R_1, R_2, R_3, R_4, and R_5 are the radiative rates of $^4I_{13/2}$, $^4I_{11/2}$, $^4I_{9/2}$, $^4F_{9/2}$ and ($^2H_{11/2}$+$^4S_{3/2}$) levels of Er^{3+}, respectively. R'_{ij} is non-radiative rate from level i to level j. W is the rate of the energy transfer process between Er^{3+}. ρ is the laser photon number density. σ_{ij} denotes the absorption cross-section between level i and j of Er^{3+}. C_6-C_8 represent cross-relaxation processes of $^4I_{13/2}$+$^4I_{13/2}$→$^4I_{15/2}$+$^4I_{9/2}$, $^4I_{13/2}$+$^4I_{11/2}$→$I_{15/2}$+$^4F_{9/2}$, and $^4I_{13/2}$+$^4I_{9/2}$ → $^4I_{15/2}$+$^4S_{3/2}$. Taking into account that the excited state populations (<10^{16} ions/cm^3) are a small fraction of all the Er^{3+} (N_{Er}>10^{20} ions/cm^3), it can be assumed that $N_0 \approx N_{Er}$. At steady state (dN_i/dt=0), the rate equations can be simplified as:

$$\sigma_{01}\rho N_0 - \sigma_{13}\rho N_1 - C_8N_1N_3 - R_1N_1 = 0 \quad (19)$$

$$R'_{32}N_3 - \sigma_{24}\rho N_2 - R_2N_2 = 0 \quad (20)$$

$$\sigma_{13}\rho N_1 - \sigma_{35}\rho N_3 - R'_{32}N_3 - C_8N_1N_3 - R_3N_3 = 0 \quad (21)$$

$$\sigma_{24}\rho N_2 - R_4N_4 = 0 \quad (22)$$

$$\sigma_{35}\rho N_3 + C_8N_1N_3 - R_5N_5 = 0 \quad (23)$$

$$N_{Er} = N_0 \quad (24)$$

The intensities of green emissions (I_{green}) can be given according to the population densities

of N_5:

$$I_{green} = N_5 R_5 \propto \frac{\sigma_{13}^2 \sigma_{35} \rho^3}{C_8 (R_1 + \delta_{13}\rho)(R_3 + R'_{32} + \sigma_{35}\rho)} \quad (25)$$

Supplementary Note 5 - Determination of PDs's performance

Three representative parameters to characterize the performance of the PDs including photo-responsivity (*R*), detectivity (*D**) and external quantum efficiency (*EQE*) (Supplementary Ref. 3). *R* represents the photocurrent (I_{ph}) generated per unit of incident power, *D** is the ability of detecting weak signal from a noisy environment, and *EQE* presents the number of carriers produced in the external circuit for each absorbed incident photon, respectively. They are defined by the following equations:

$$R = \frac{I_{\mathrm{light}} - I_{\mathrm{dark}}}{PS} \,, \quad (26)$$

$$\mathrm{D}^* = \frac{\mathrm{R}}{(2\mathrm{eI}_{\mathrm{dark}}/\mathrm{S})^{\frac{1}{2}}} \,, \quad (27)$$

$$\mathrm{EQE} = \mathrm{R}\frac{hc}{\lambda \mathrm{e}} \,, \quad (28)$$

where I_{light} and I_{dark} are the photocurrents of PDs separately under the light illumination and in the dark; *P* is the input light power intensity, *S* is the effective illuminated area; *h, c,* λ, and *e* represents the Planck's constant, the velocity of light, the wavelength of incident light, and the elementary charge, respectively.

II. Supplementary Figures

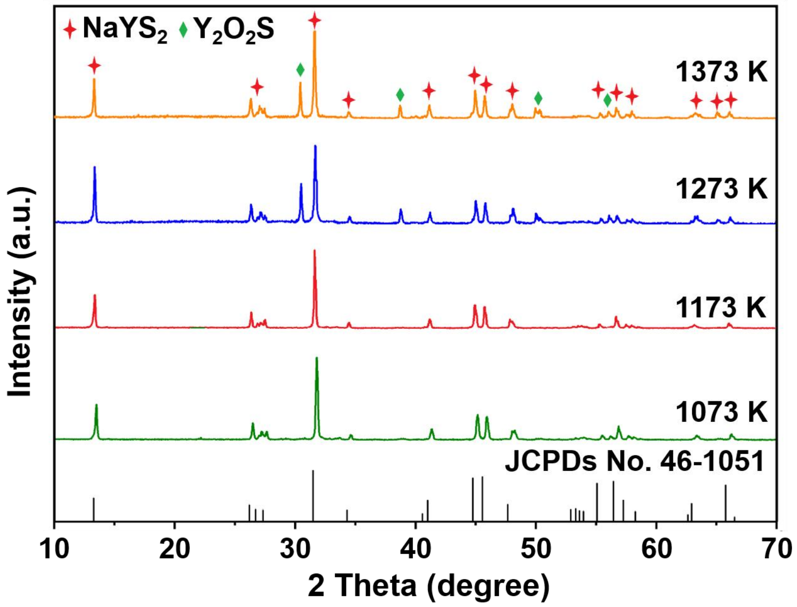

Supplementary Figure 3. Phase structure characterization of $NaYS_2$:Er^{3+} synthesized at different temperatures. XRD patterns of $NaYS_2$:Er^{3+} prepared at 1073, 1173, 1273, and 1373 K. The samples prepared at 1073 and 1173 K are trigonal phase $NaYS_2$, there are two phases of Y_2O_2S and $NaYS_2$ while the synthesis temperature of 1273, and 1373 K.

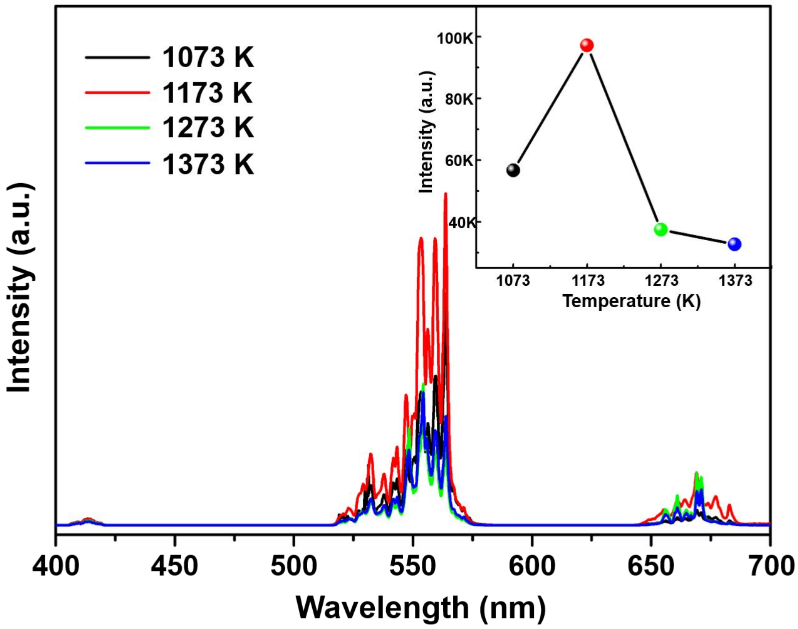

Supplementary Figure 4. UC luminescence of $NaYS_2$:Er^{3+} synthesized at different temperatures. UC luminescence spectra of $NaYS_2$:Er^{3+} phosphors prepared at 1073, 1173, 1273, and 1373 K under 1532 nm excitation (inset: integral intensity of $NaYS_2$:Er^{3+} varies with synthesis temperature). The luminescence intensity of the samples presents a tendency to increase first and then decrease with the temperature rises.

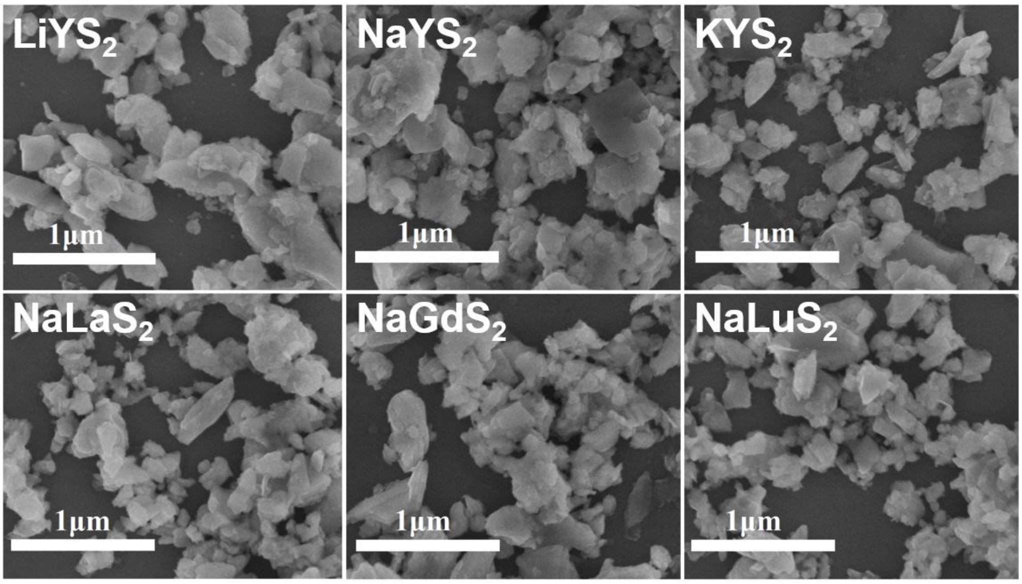

Supplementary Figure 5. Morphology characterization of $MLnS_2$. SEM images of $MLnS_2$ (M=Li, Na, K; Ln=La, Y, Lu, Gd) phosphors, the samples present irregular morphology.

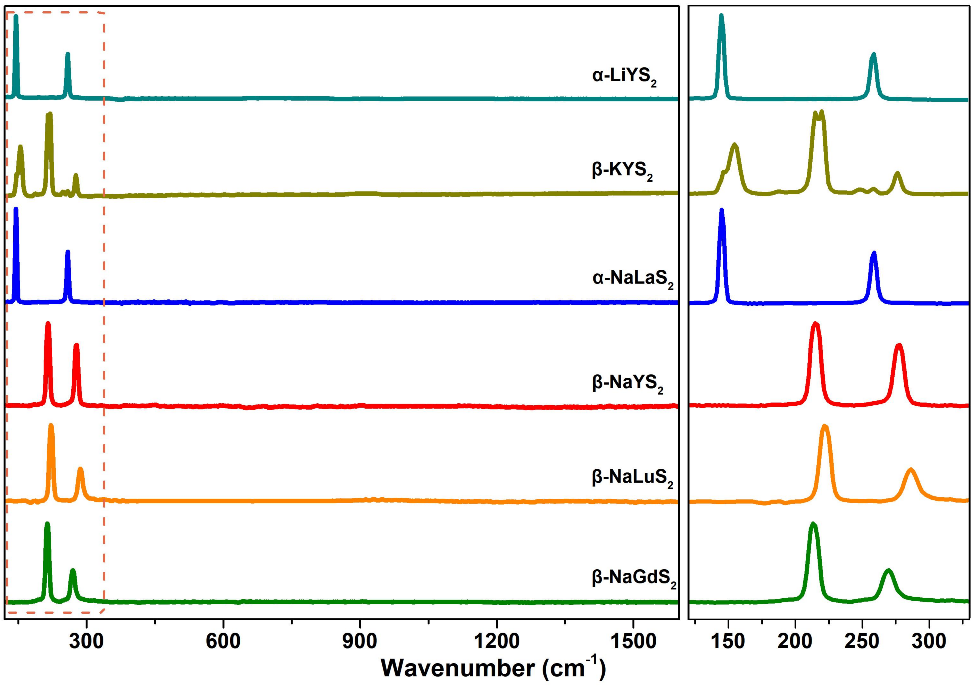

Supplementary Figure 6. Phonon energy of $MLnS_2$. Raman spectra of $MLnS_2$ (M=Li, Na, K; Ln=La, Y, Lu, Gd) hosts, the phonon energies of all the samples are lower than 300 cm^{-1}.

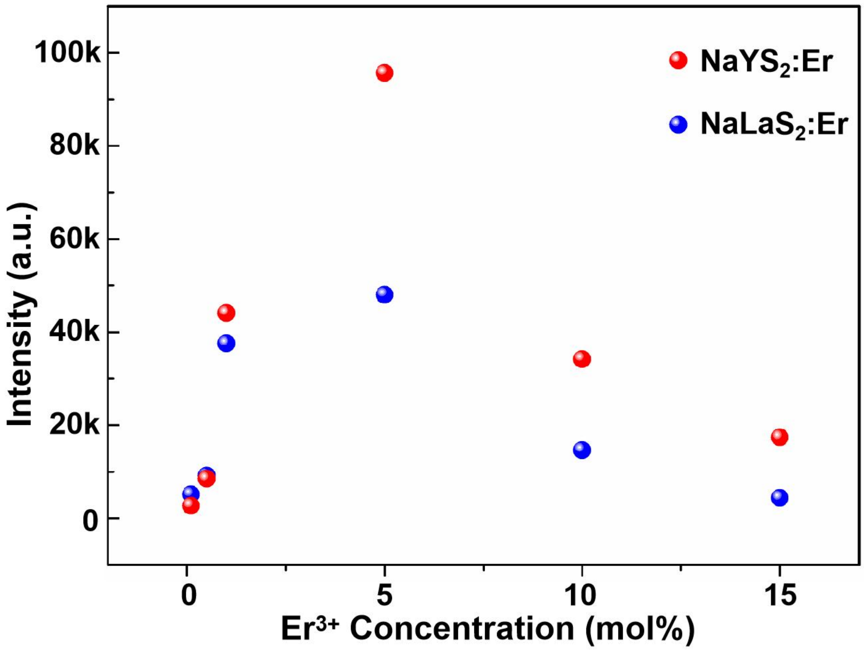

Supplementary Figure 7. UC luminescence intensity of NaLaS$_2$:Er^{3+} and NaYS$_2$:Er^{3+}. Integral intensity of NaLaS$_2$:xEr^{3+} and NaYS$_2$:xEr^{3+} (x=0.1, 0.5, 2, 5, 10, 15%) under 1532 nm excitation at 1.45 W cm^{-2}.

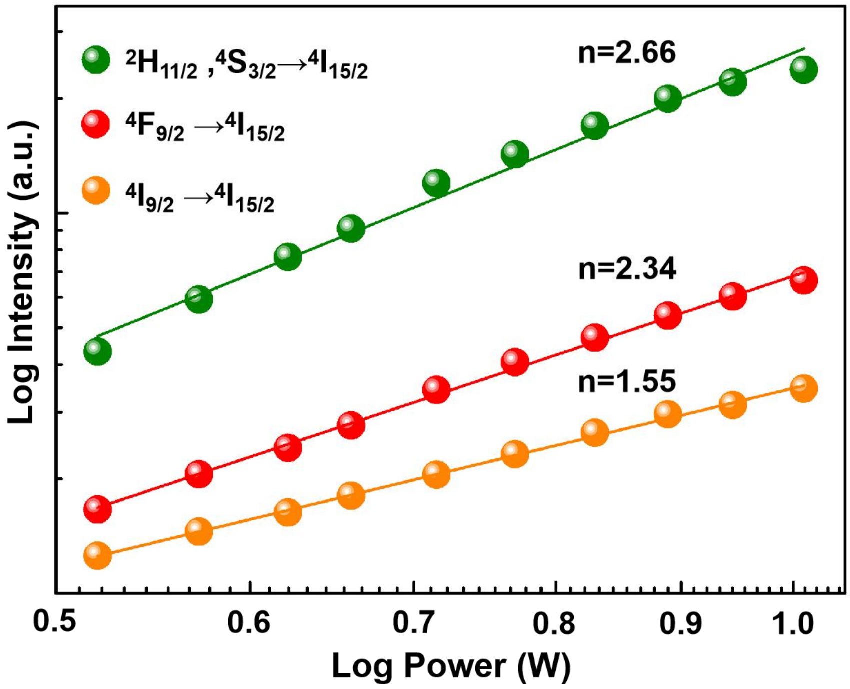

Supplementary Figure 8. Power dependent UC emissions. Dependence of the green, red and NIR integral UC emission intensities of NaYS$_2$:Er^{3+} on the excitation power of 1532 nm, which are three-, three-, and two-photon UCs, respectively.

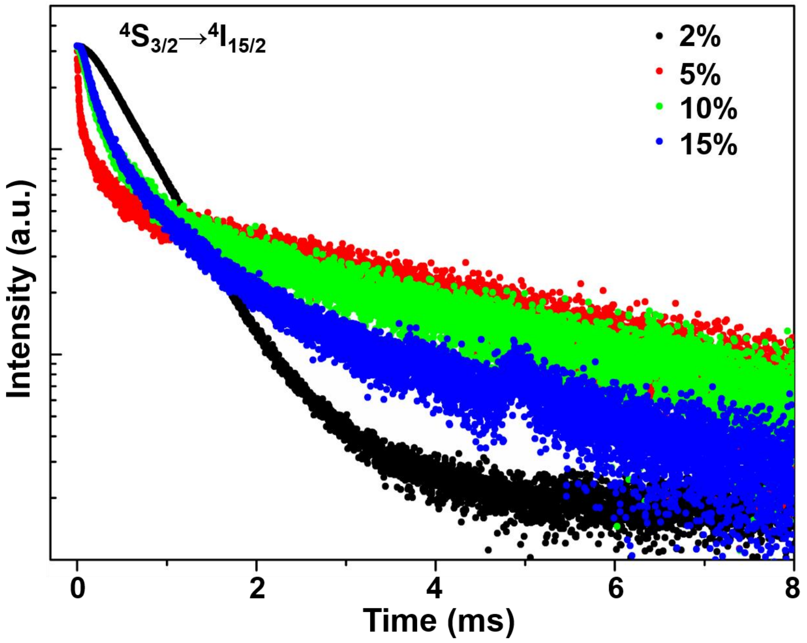

Supplementary Figure 9. Decay lifetimes of $^4S_{3/2}\rightarrow{}^4I_{15/2}$ level. Decay profiles of 564 nm (corresponding to the $^4S_{3/2}\rightarrow{}^4I_{15/2}$ transition) under 1532 nm excitation for $NaYS_2$:xEr^{3+} (x= 2, 5, 10, 15 mol%). The lifetime values increase rapidly from 0.87 ms to 3.37 ms when Er^{3+} concentration increases from 2 to 5 mol%, and then decrease as the concentration continues increasing to 15 mol%, which might illustrate that a concentration quenching effect occurs while the Er^{3+} concentration higher than 5 mol%.

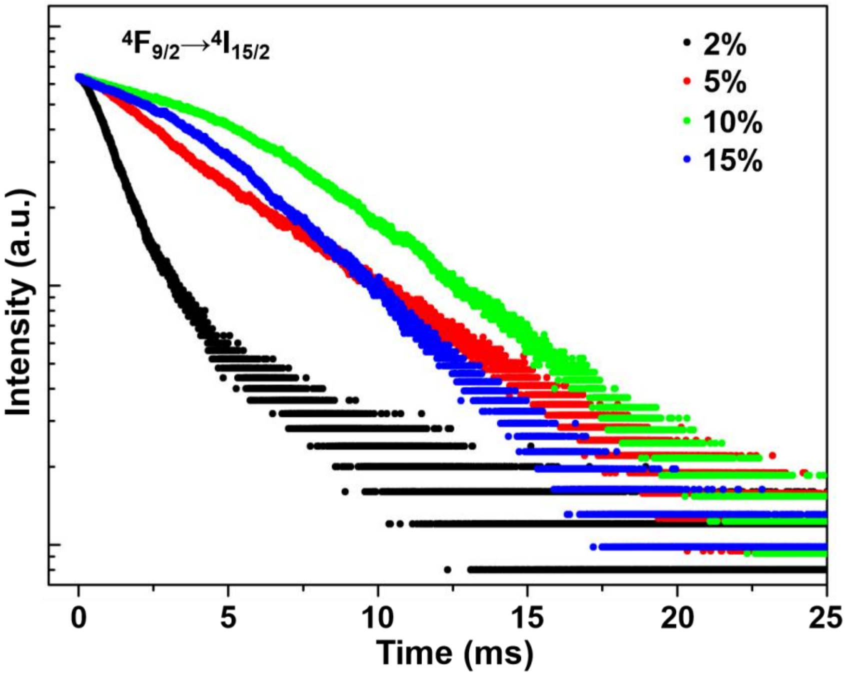

Supplementary Figure 10. Decay lifetimes of $^4F_{9/2}\rightarrow{}^4I_{15/2}$ level. Decay profiles of 660 nm (corresponding to the $^4F_{9/2}\rightarrow{}^4I_{15/2}$ transition) under 1532 nm excitation for $NaYS_2$:xEr^{3+} (x= 2, 5, 10, 15 mol%), the lifetimes of the $^4F_{9/2}$ state are evaluated to be 1.82-6.94 ms.

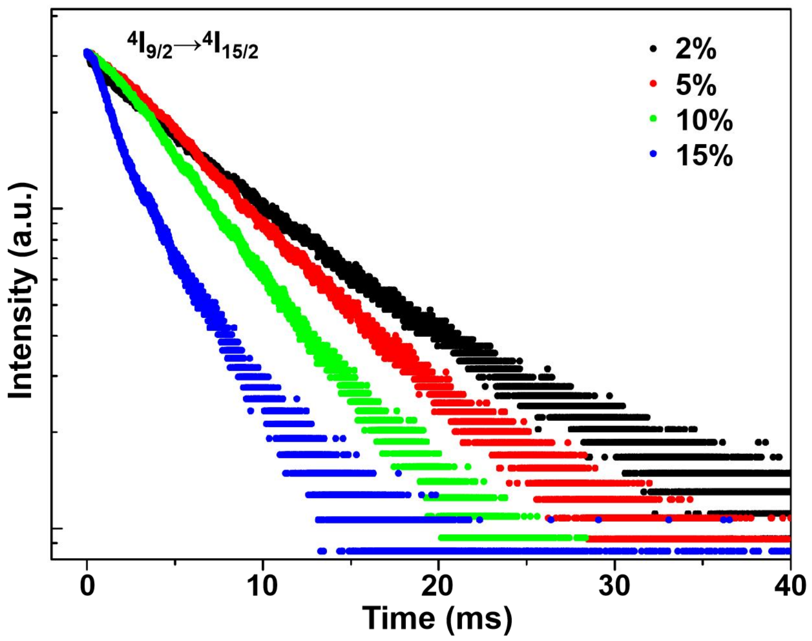

Supplementary Figure 11. Decay lifetimes of $^4I_{9/2}\rightarrow^4I_{15/2}$ level. Decay profiles of 800 nm (corresponding to the $^4I_{9/2}\rightarrow^4I_{15/2}$ transition) under 1532 nm excitation for $NaYS_2$:xEr^{3+} (x= 2, 5, 10, 15 mol%), the lifetimes of the $^4I_{9/2}$ state are evaluated to be 4.8-10.28 ms.

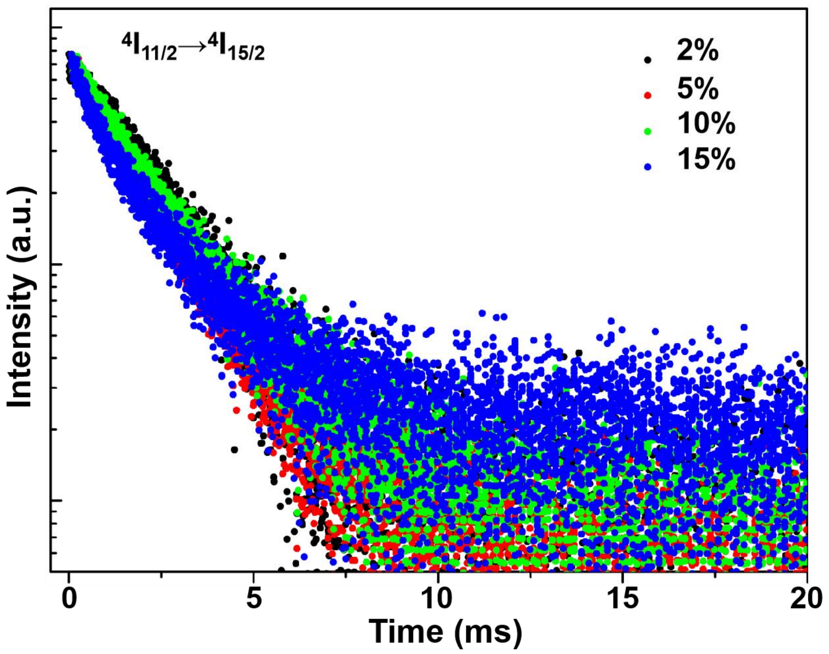

Supplementary Figure 12. Decay lifetimes of $^4I_{11/2}\rightarrow^4I_{15/2}$ level. Decay profiles of 1000 nm (corresponding to the $^4I_{11/2}\rightarrow^4I_{15/2}$ transition) under 1532 nm excitation for $NaYS_2$:xEr^{3+} (x= 2, 5, 10, 15 mol%).

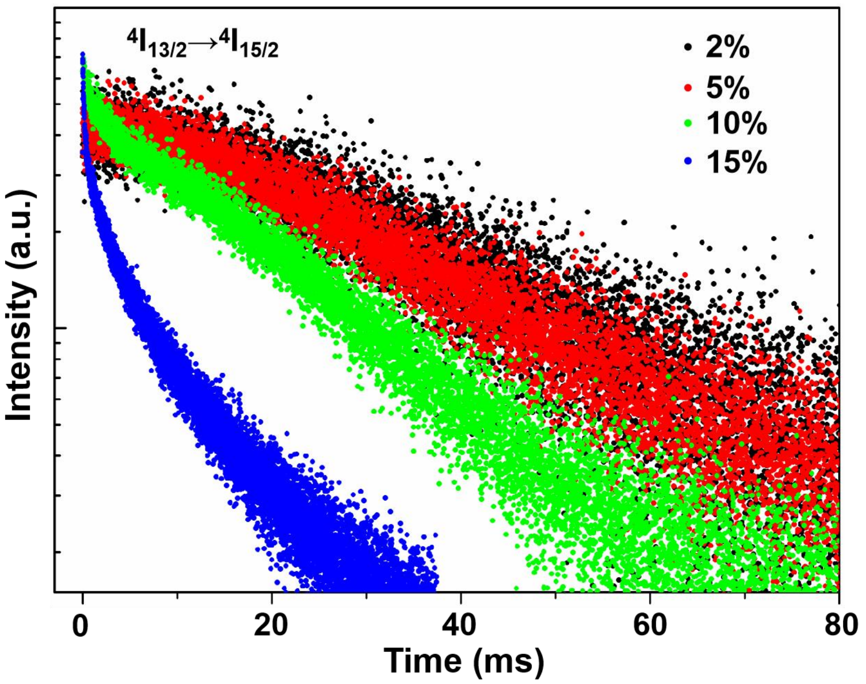

Supplementary Figure 13. Decay lifetimes of $^4I_{13/2}$→$^4I_{15/2}$ level. Decay profiles of 1500 nm (corresponding to the $^4I_{13/2}$→$^4I_{15/2}$ transition) under 980 nm excitation for $NaYS_2$:xEr^{3+} (x= 2, 5, 10, 15 mol%). $NaYS_2$:Er^{3+} phosphors present ultra-long lifetimes of 9.44 to 33.04 ms, respectively.

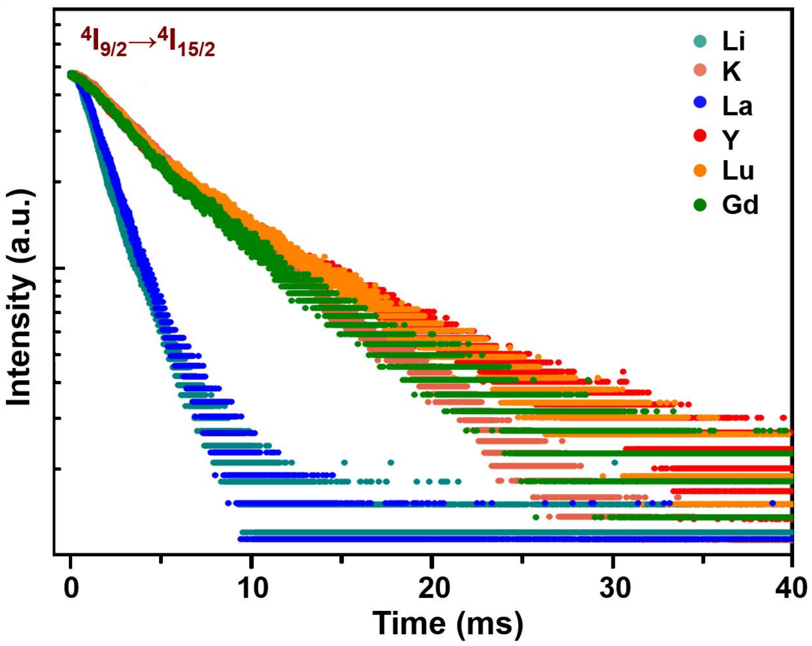

Supplementary Figure 14. Decay lifetimes of $^4I_{9/2}$→$^4I_{15/2}$ level in $MLnS_2$:Er^{3+}. Decays curves of $^4I_{9/2}$→$^4I_{15/2}$ transition of Er^{3+} in $MLnS_2$:Er^{3+} (M=Li, Na, K; Ln=La, Y, Lu, Gd).

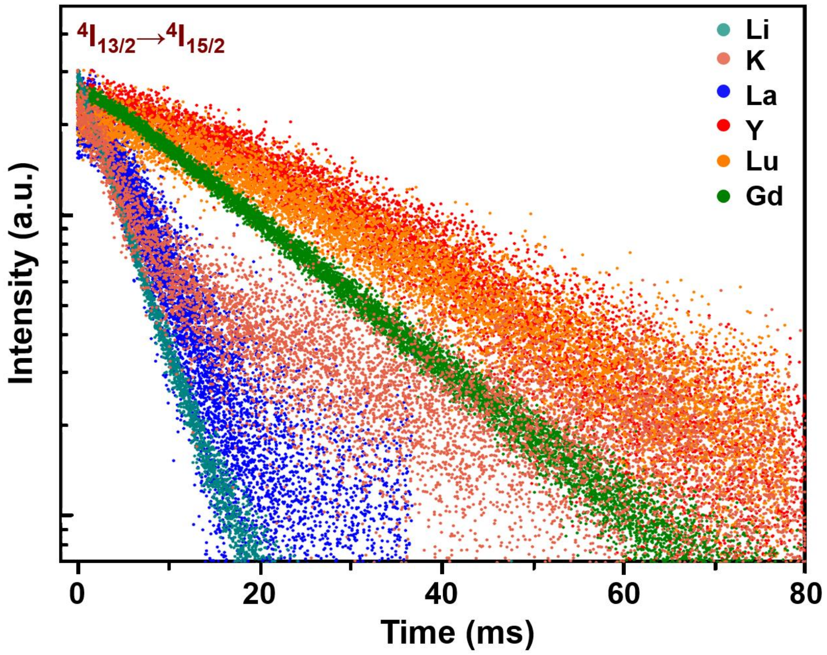

Supplementary Figure 15. Decay lifetimes of $^4I_{13/2}\rightarrow^4I_{15/2}$ level in $MLnS_2:Er^{3+}$. Decays curves of $^4I_{13/2}\rightarrow^4I_{15/2}$ transition of Er^{3+} in $MLnS_2:Er^{3+}$ (M=Li, Na, K; Ln=La, Y, Lu, Gd).

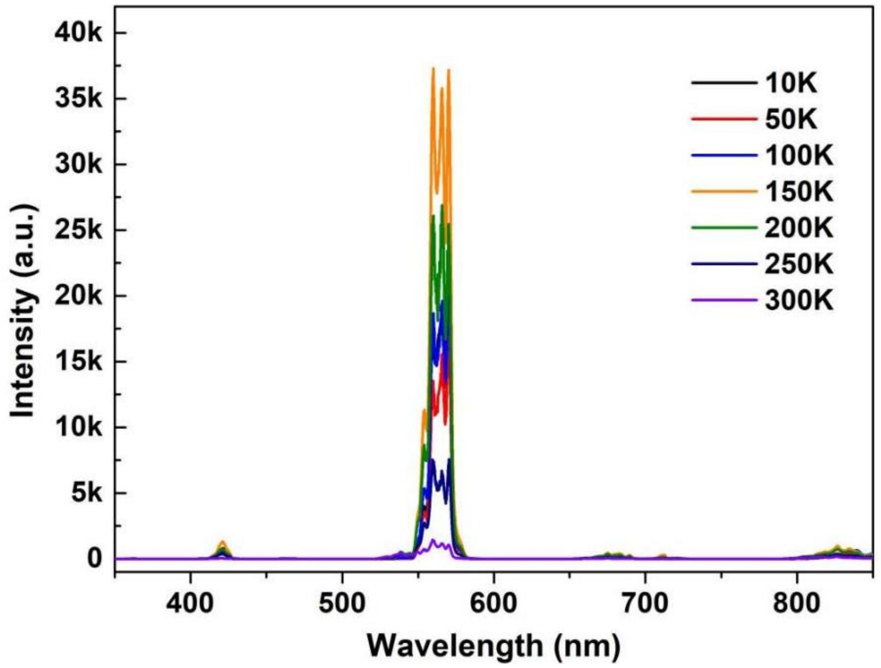

Supplementary Figure 16. Temperature dependent UC luminescence intensity. UC luminescence spectra of $NaYS_2:Er^{3+}$ under 1532 nm excitation at verified temperatures from 10-300 K.

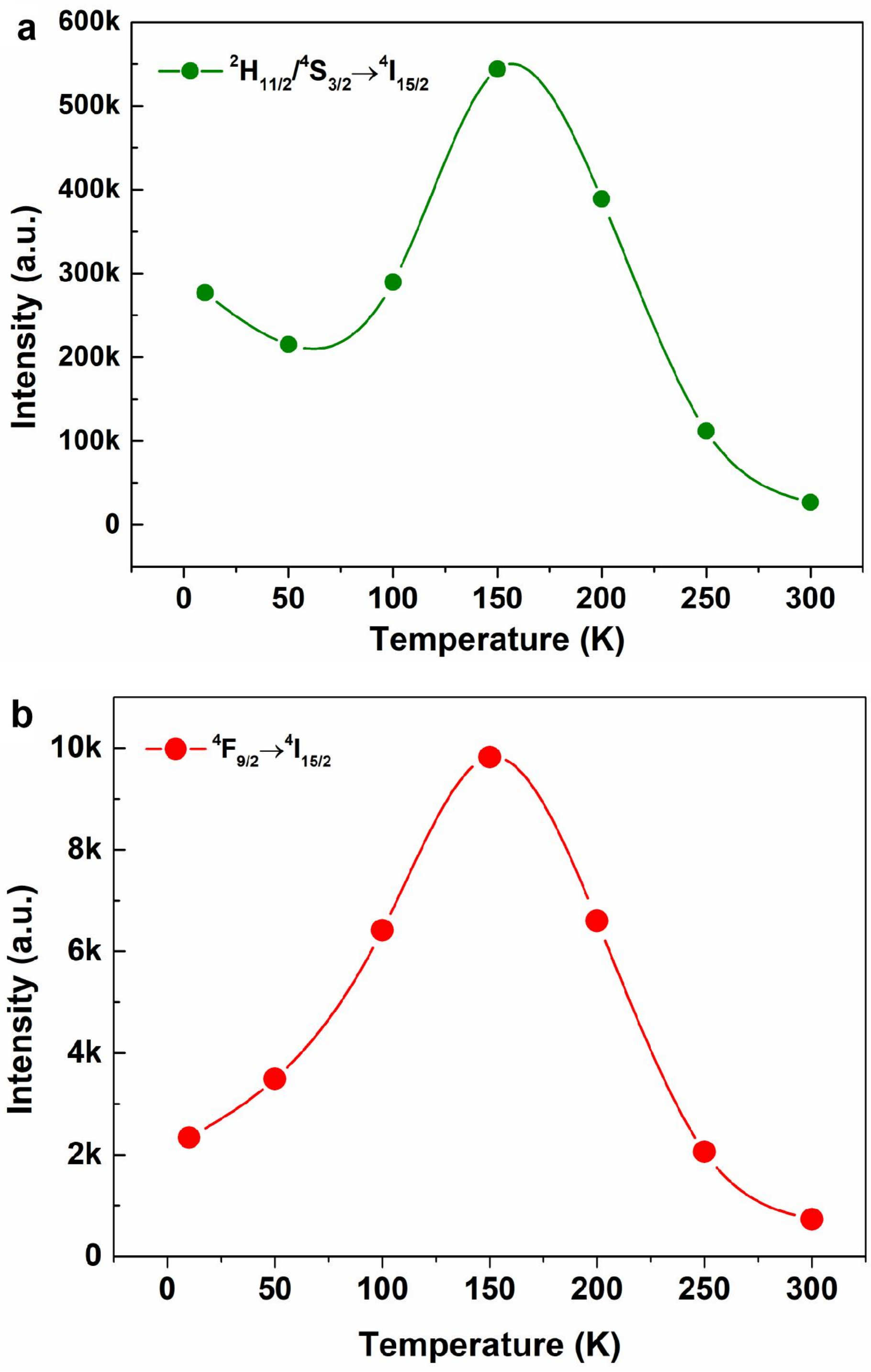
a
$^2H_{11/2}/^4S_{3/2}\rightarrow^4I_{15/2}$
Intensity (a.u.)
600k
500k
400k
300k
200k
100k
0
0
50
100
150
200
250
300
Temperature (K)
b
$^4F_{9/2}\rightarrow^4I_{15/2}$
Intensity (a.u.)
10k
8k
6k
4k
2k
0
0
50
100
150
200
250
300
Temperature (K)

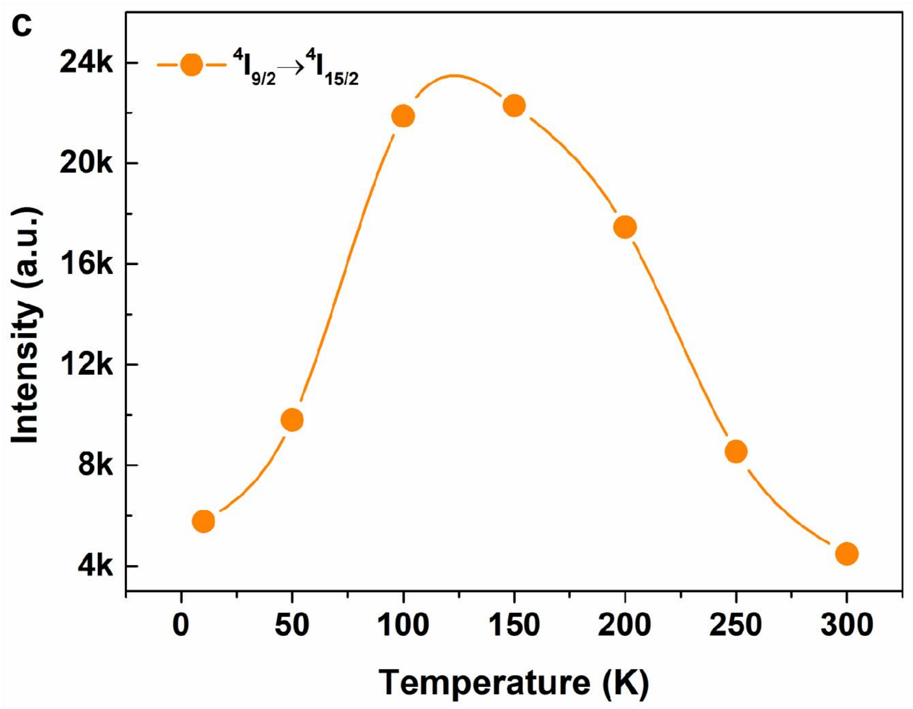

Supplementary Figure 17. Temperature dependent UC luminescence integral intensity. Integral intensity of (a) $^2H_{11/2}/^4S_{3/2}\rightarrow{}^4I_{15/2}$, (b) $^4F_{9/2}\rightarrow{}^4I_{15/2}$, (c) $^4I_{9/2}\rightarrow{}^4I_{15/2}$ of $NaYS_2:Er^{3+}$ under 1532 nm excitation at verified temperatures from 10-300 K in vacuum.

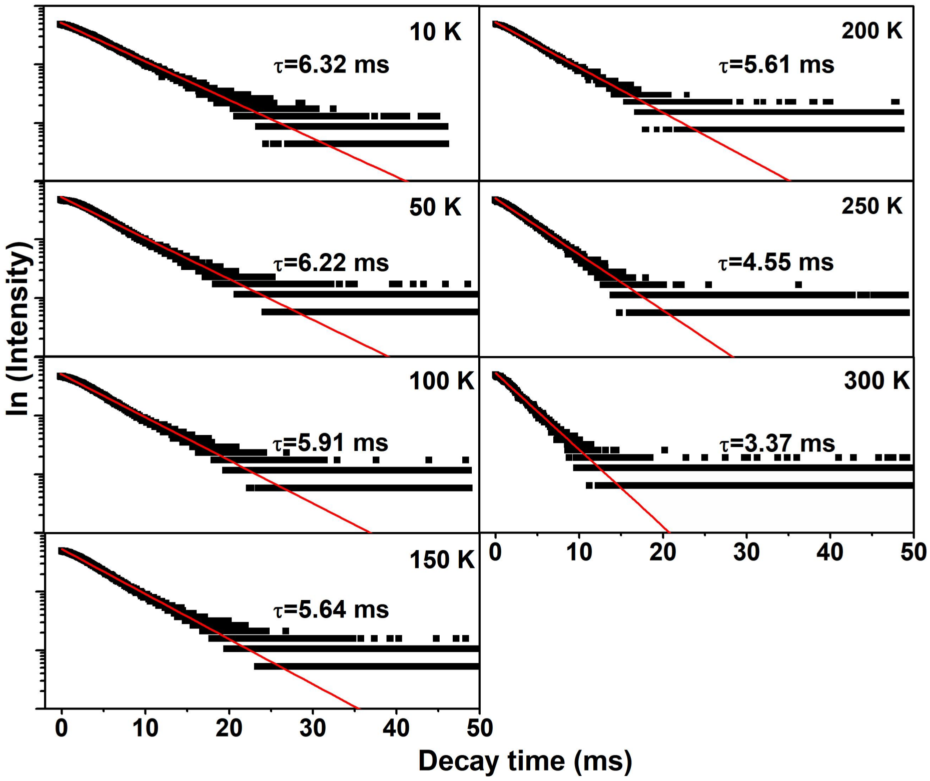

Supplementary Figure 18. Temperature dependent decay lifetimes of $^4S_{3/2}\rightarrow{}^4I_{15/2}$. UC luminescence decay times of $^4S_{3/2}\rightarrow{}^4I_{15/2}$ transitions of Er^{3+} in $NaYS_2:Er^{3+}$ with different temperature.

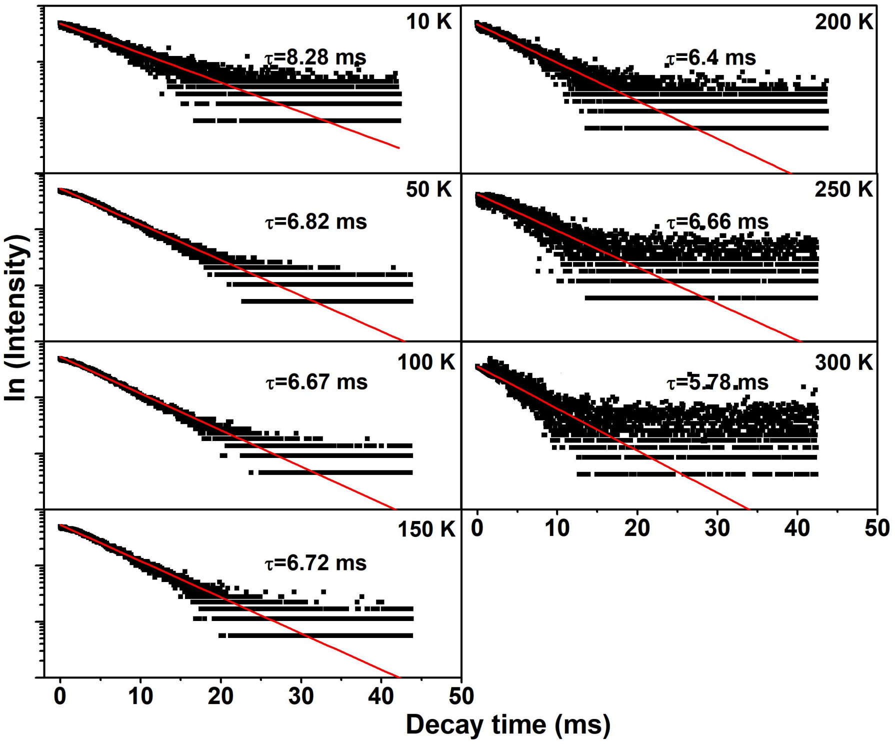

Supplementary Figure 19. Temperature dependent luminescence lifetimes of $^4F_{9/2}\rightarrow{}^4I_{15/2}$. UC luminescence decay times of $^4F_{9/2}\rightarrow{}^4I_{15/2}$ transitions of Er^{3+} in $NaYS_2:Er^{3+}$ with different temperature.

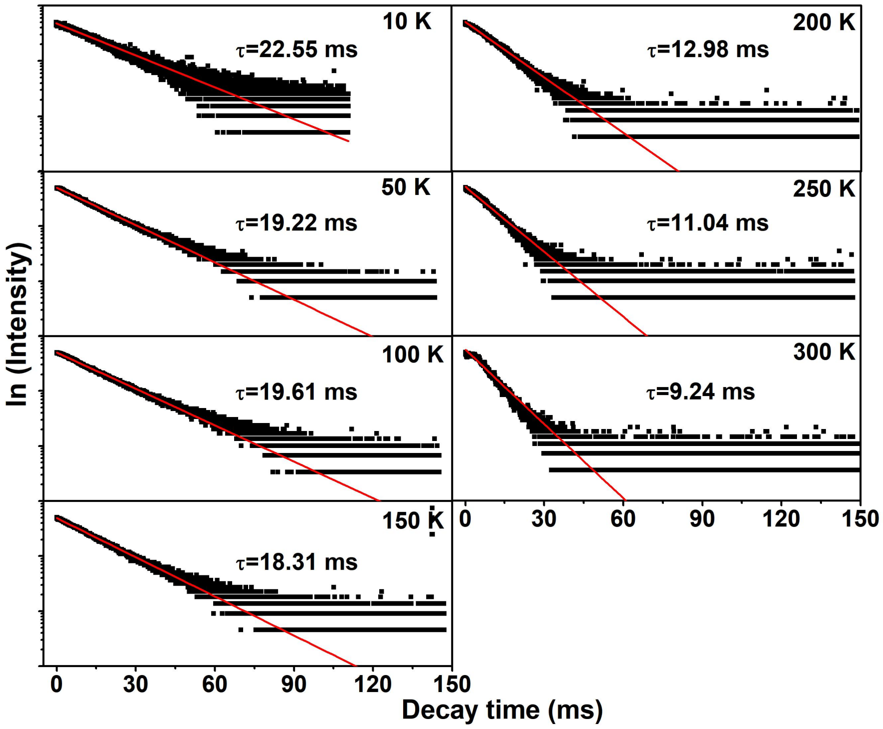

Supplementary Figure 20. Temperature dependent decay lifetimes of $^4I_{9/2}\rightarrow{}^4I_{15/2}$. UC luminescence decay times of $^4I_{9/2}\rightarrow{}^4I_{15/2}$ transitions of Er^{3+} in $NaYS_2:Er^{3+}$ with different temperature.

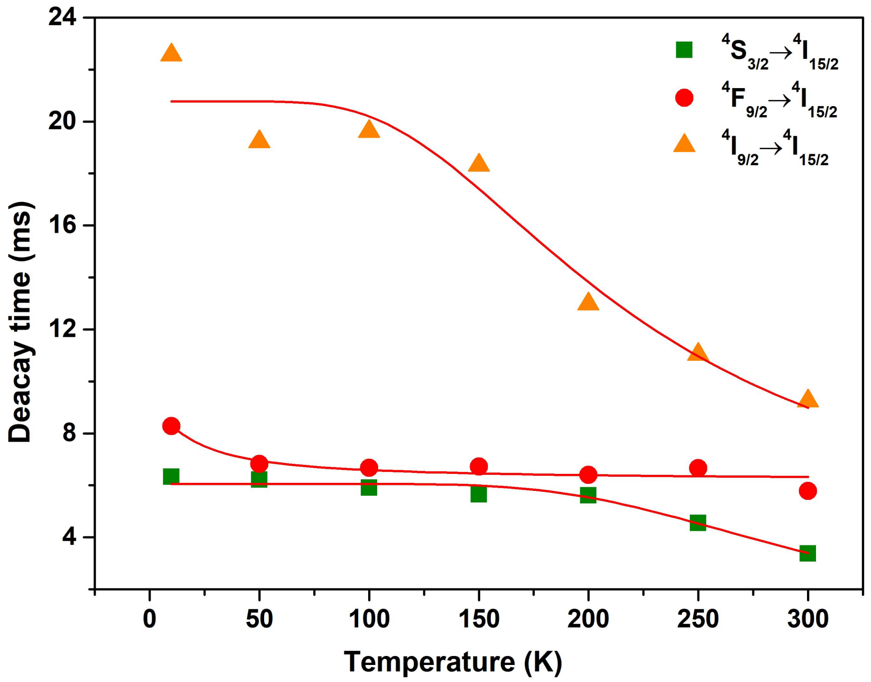

Supplementary Figure 21. Temperature dependent decay times constants. Decay time constants of $^4S_{3/2}\rightarrow{}^4I_{15/2}$, $^4F_{9/2}\rightarrow{}^4I_{15/2}$, $^4I_{9/2}\rightarrow{}^4I_{15/2}$ transitions in $NaYS_2:Er^{3+}$ as a function of temperature from 10 K to 300 K.

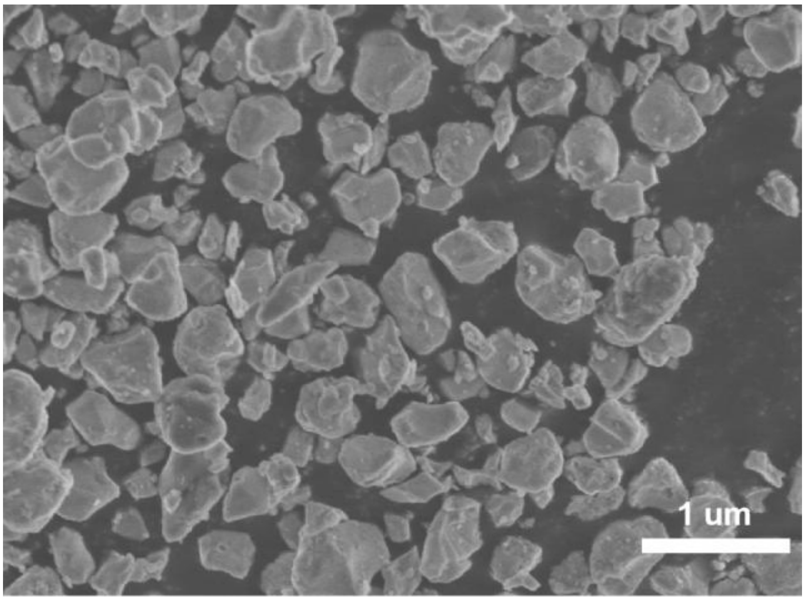

Supplementary Figure 22. Morphology characterization of $NaYF_4:Yb^{3+},Er^{3+}$. SEM image of the standard $NaYF_4:Yb^{3+},Er^{3+}$ phosphor, the sample presents irregular morphology with micron size.

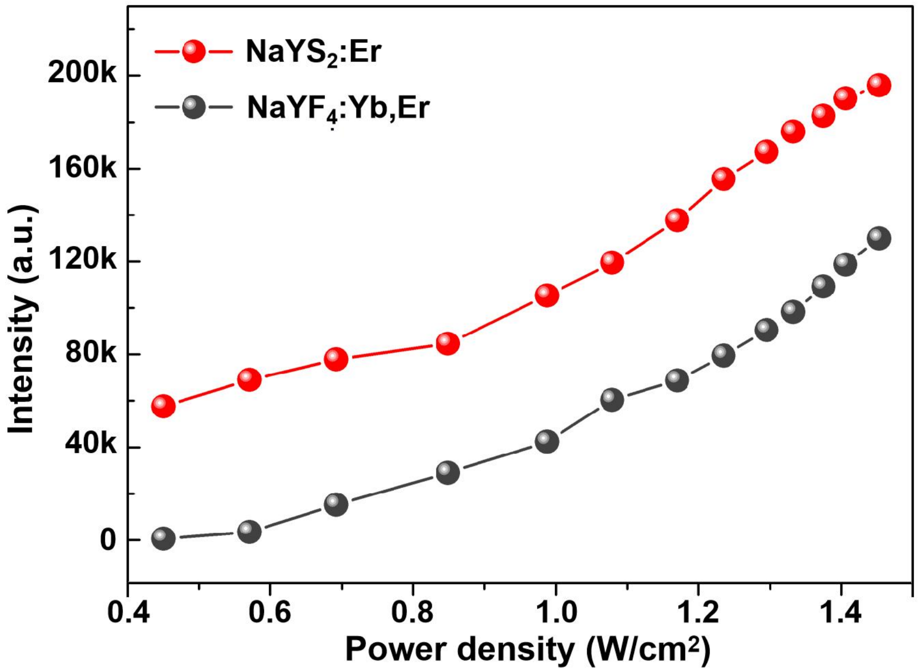

Supplementary Figure 23. Luminescence intensity comparison of $NaYS_2:Er^{3+}$ and $NaYF_4:Yb^{3+},Er^{3+}$. Integral intensity of $NaYS_2:Er^{3+}$ under 1532 nm excitation and $NaYF_4:Yb^{3+},Er^{3+}$ under 980 nm excitation with various excitation power density (W cm^{-2}).

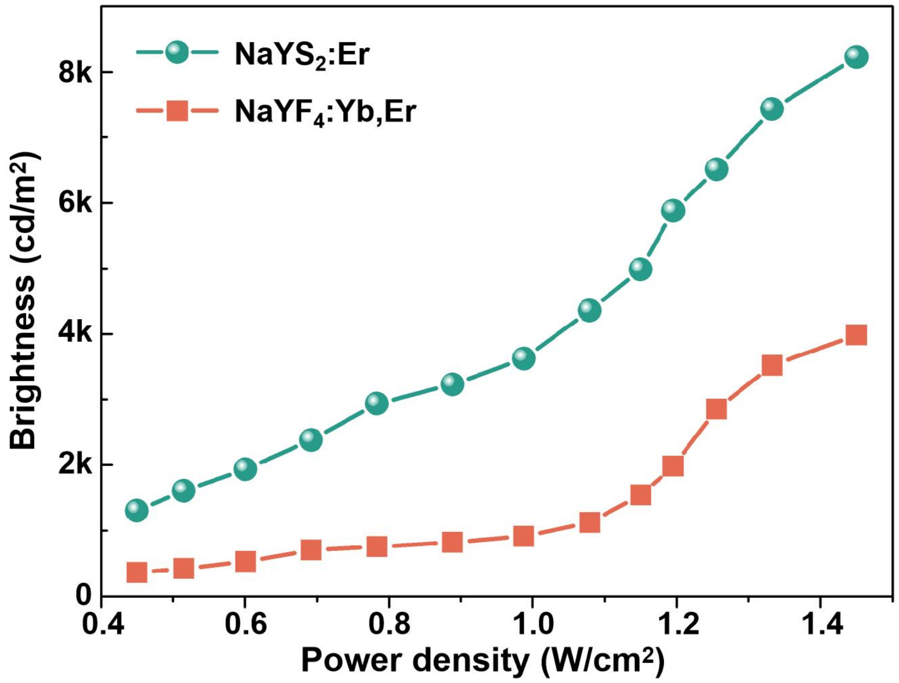

Supplementary Figure 24. Brightness comparison of $NaYS_2:Er^{3+}$ and $NaYF_4:Yb^{3+},Er^{3+}$ at same power density. Brightness of $NaYS_2:Er^{3+}$ under 1532 nm excitation and $NaYF_4:Yb^{3+},Er^{3+}$ under 980 nm excitation with various power density (W cm^{-2}).

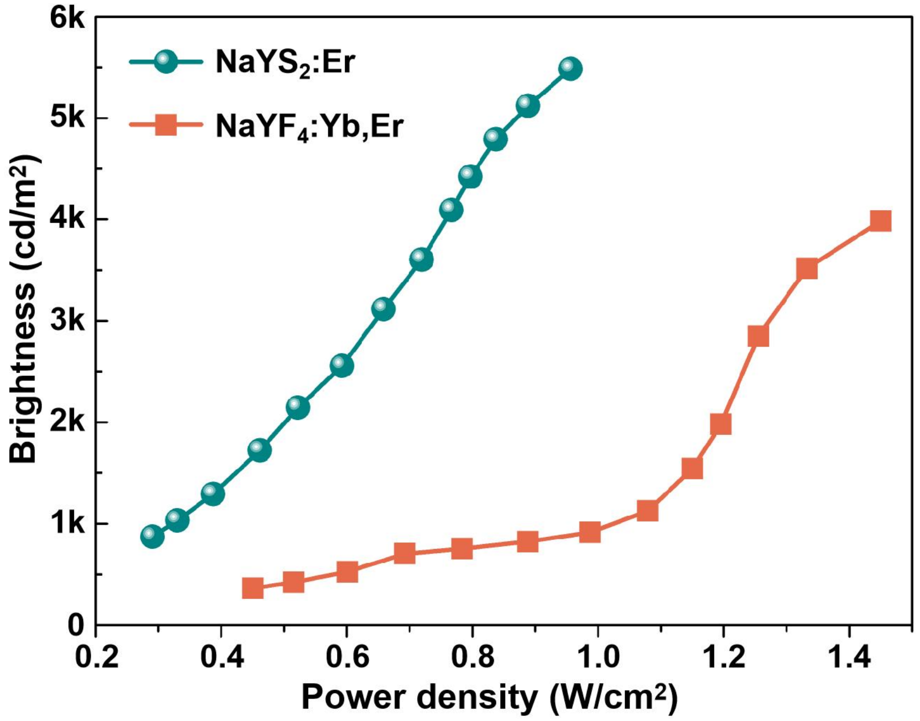

Supplementary Figure 25. Brightness comparison of $NaYS_2:Er^{3+}$ and $NaYF_4:Yb^{3+},Er^{3+}$ at same photon flux. Brightness of $NaYS_2:Er^{3+}$ under 1532 nm excitation and $NaYF_4:Yb^{3+},Er^{3+}$ under 980 nm excitation with various power density (W cm^{-2}) while maintains the same photon flux.

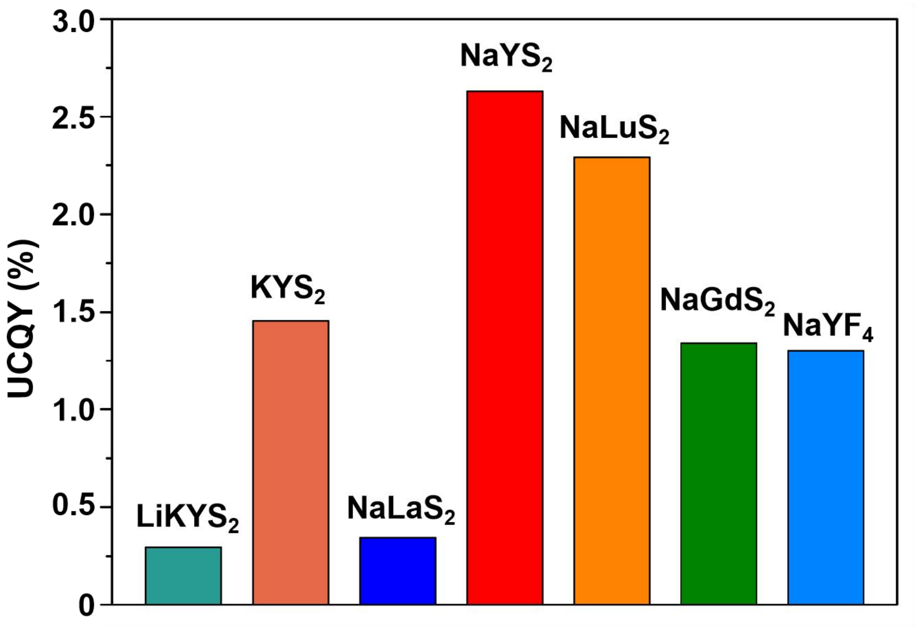

Supplementary Figure 26. UCQYs of $MLnS_2:Er^{3+}$. UCQYs of $MLnS_2:Er^{3+}$ (M=Li, Na, K; Ln=La, Y, Lu, Gd) under 1532 nm excitation and $NaYF_4:Yb^{3+},Er^{3+}$ under 980 nm excitation (4.5 W cm^{-2}).

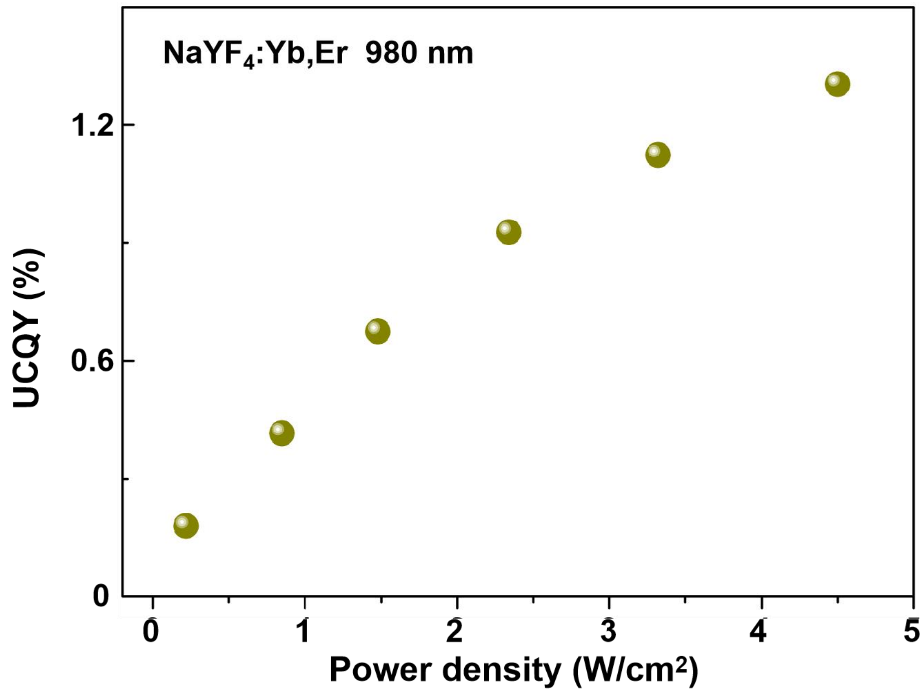

Supplementary Figure 27. UCQYs of $NaYF_4$:Yb^{3+},Er^{3+}. UCQYs of $NaYF_4$:Yb^{3+},Er^{3+} under 980 nm excitation at different power densities.

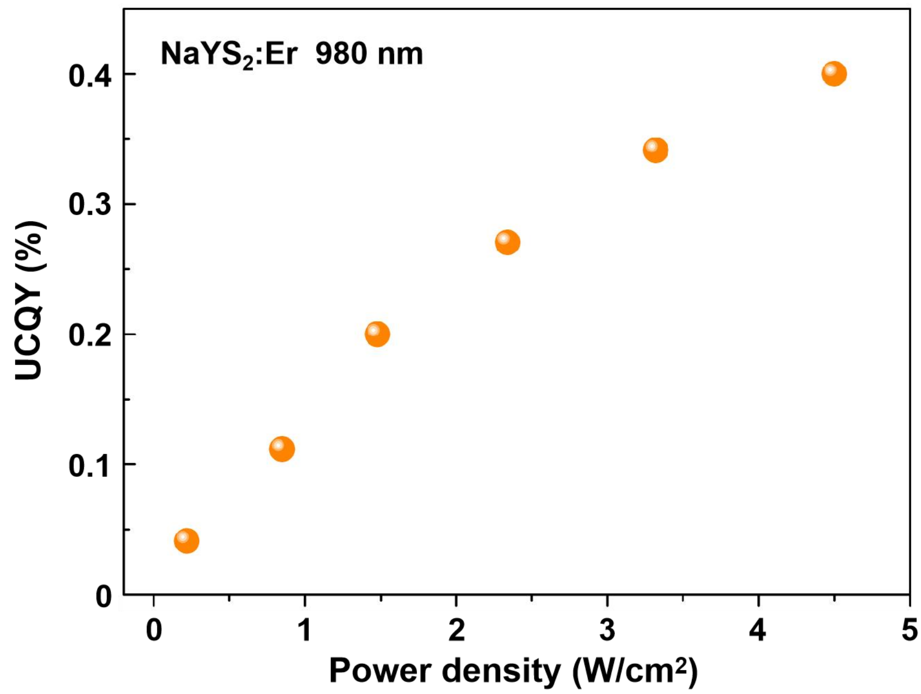

Supplementary Figure 28. UCQYs of $NaYS_2$:Er^{3+} at 980 nm irradiation. UCQYs of $NaYS_2$:Er^{3+} under 980 nm excitation at different power densities.

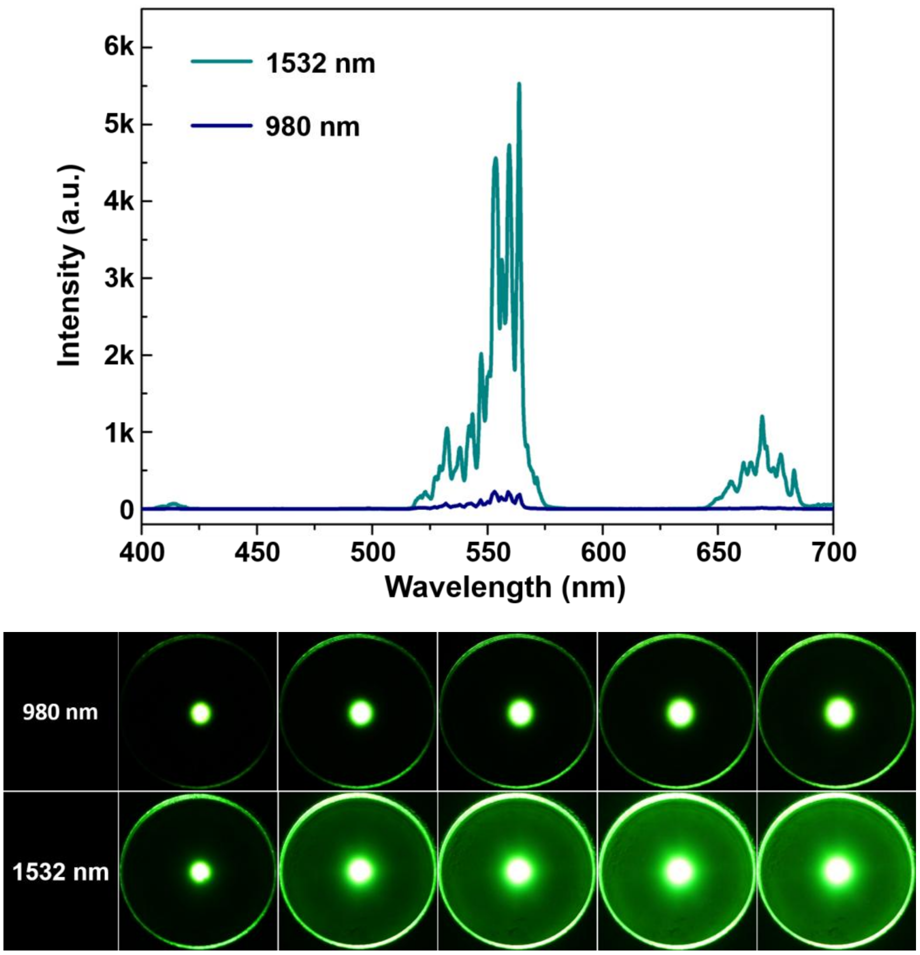

Supplementary Figure 29. UC luminescence of $NaYS_2:Er^{3+}$ with different wavelength irradiation. UC luminescence spectra and photos of $NaYS_2:Er^{3+}$ under 980 nm and 1532 nm excitation with the power density of 0.45W cm^{-2}.

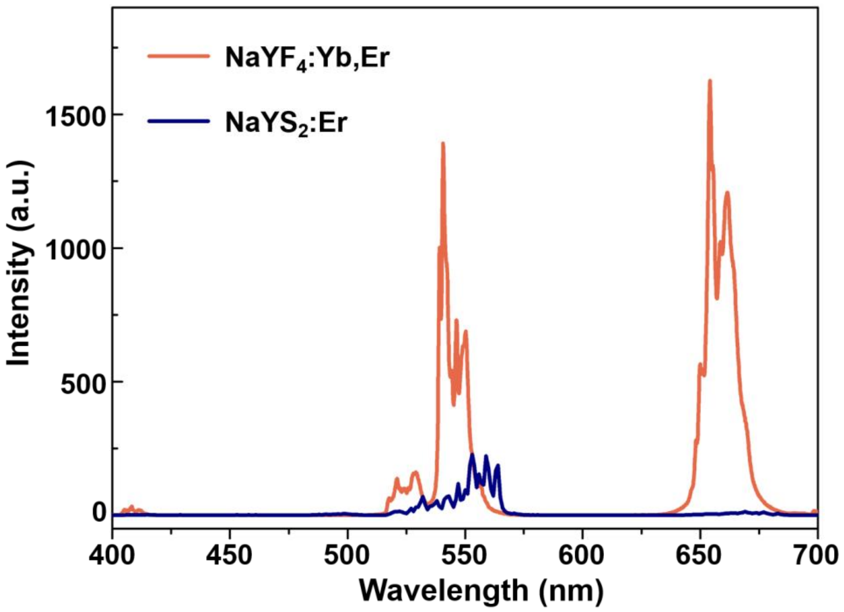

Supplementary Figure 30. UC emission of $NaYS_2:Er^{3+}$ and $NaYF_4:Yb^{3+},Er^{3+}$ with same wavelength irradiation. UC luminescence spectra of $NaYS_2:Er^{3+}$ and $NaYF_4:Yb^{3+},Er^{3+}$ under 980 nm with the power density of 0.45W cm^{-2}.

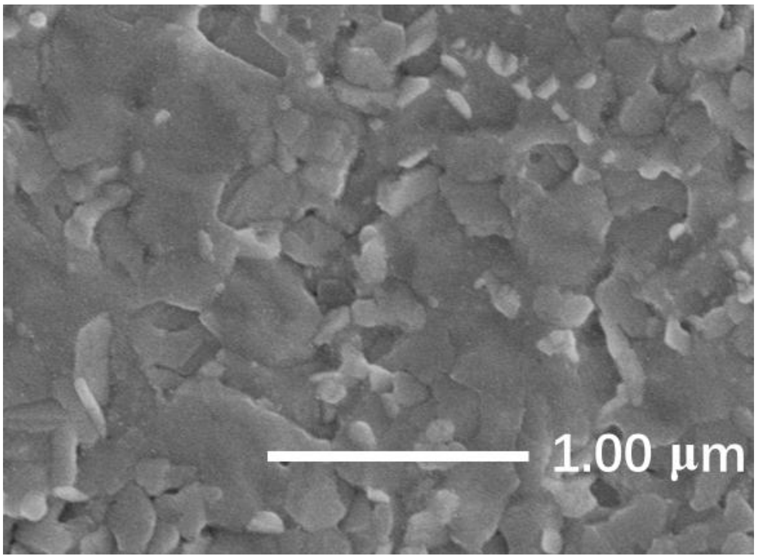

Supplementary Figure 31. Morphology characterization of $NaYS_2$:Er^{3+}/$MAPbI_3$ hybrid. Top view SEM image of the $NaYS_2$:Er^{3+}/$MAPbI_3$ hybrid.

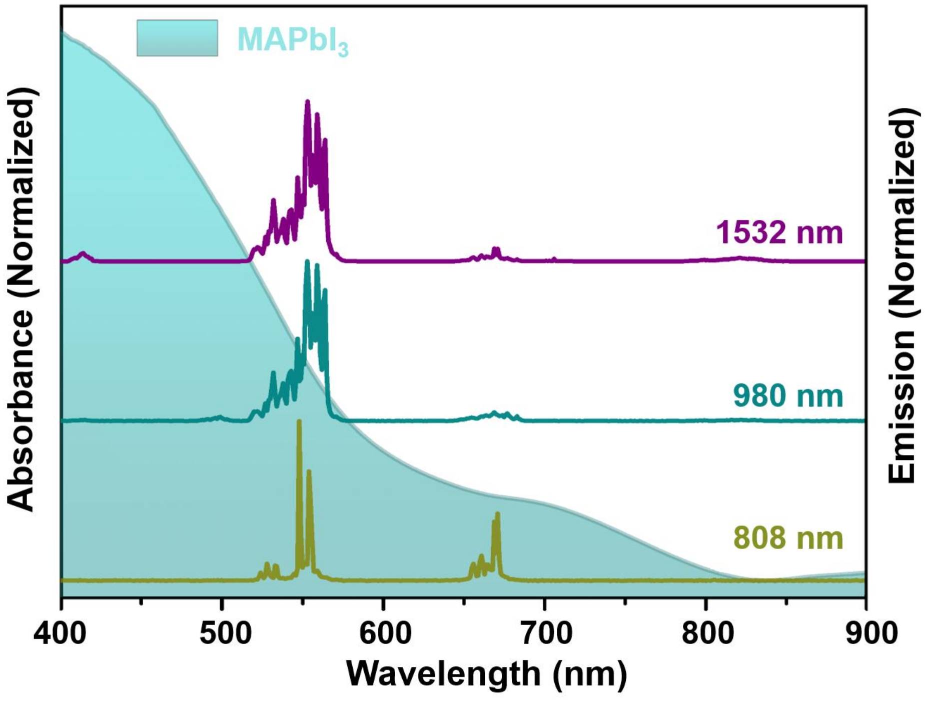

Supplementary Figure 32. Working mechanism of NIR photodetection. Absorption spectrum of $MAPbI_3$ films as well as emission spectra of $NaYS_2$:Er^{3+} phosphor from $^2H_{9/2}$, $^2H_{11/2}$/$^4S_{3/2}$, $^4F_{9/2}\rightarrow{}^4I_{15/2}$ transitions under 808, 980, and 1532 nm excitation, respectively. The phosphors absorb, and convert the NIR photons peaking around 808, 980, and 1532 nm to visible light in the spectral range of 400-700 nm.

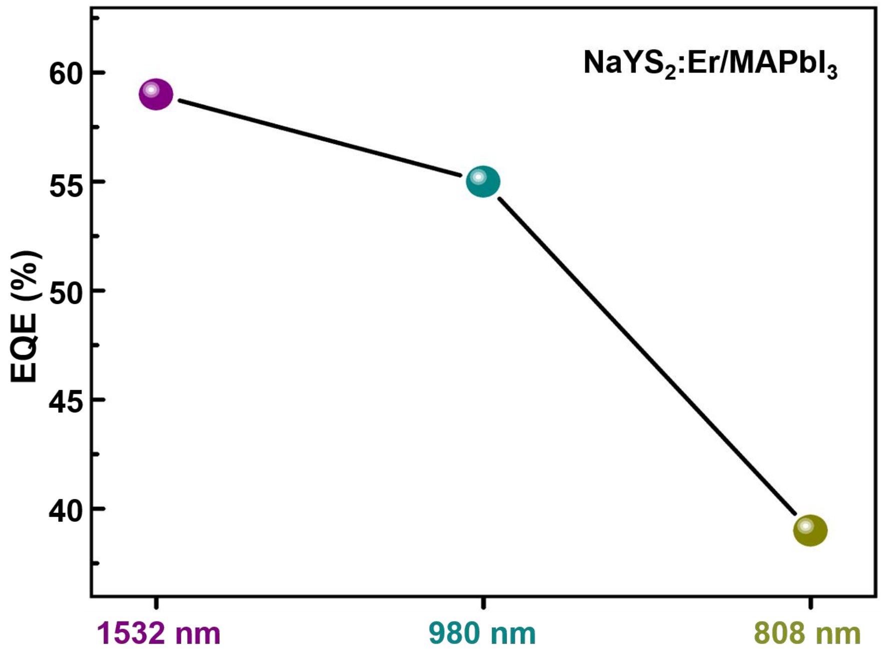

Supplementary Figure 33. EQE characterization. EQE of the NaYS$_2$:Er^{3+}/MAPbI$_3$ PD.

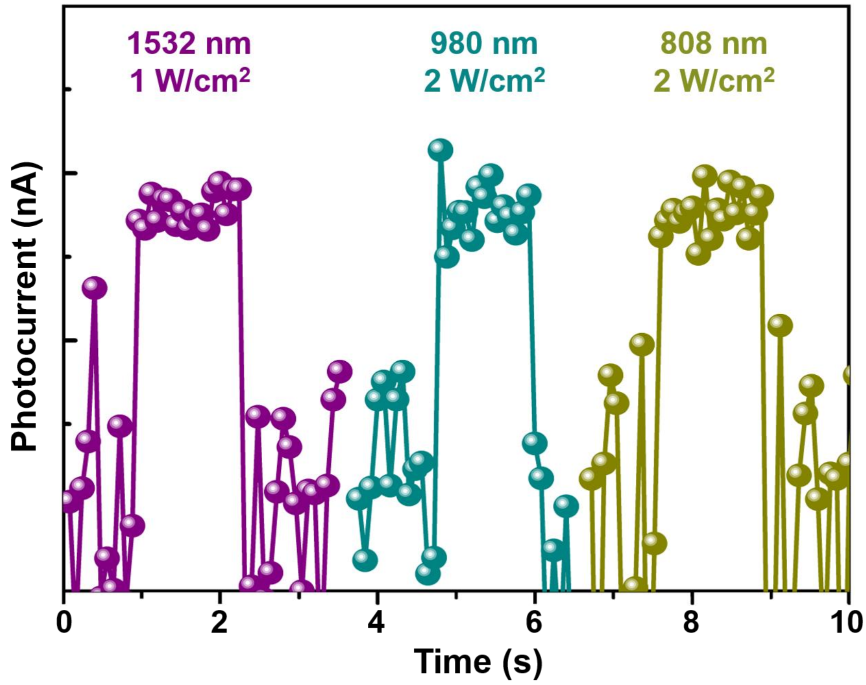

Supplementary Figure 34. On-off switching currents characterization. On-off switching currents at the lowest detectable excitation power density of NaYS$_2$:Er^{3+}/MAPbI$_3$ device under 808, 980, and 1532 nm excitation, respectively.

III. Supplementary Tables

Supplementary Table 1 Comparison of $NaYS_2$ and $NaYF_4$ from structural chemical perspective.

	$NaYS_2$	$NaYF_4$
Crystal system	Trigonal	Hexagonal
Space group	R3m	P63/m
Cell paremeters	a=b=3.9604(12)Å<br>c=19.867 (8)Å	a=b=5.6022(2) Å<br>c=3.3295(2) Å
c/a	5.016	0.594
Z	3	2
Coordination number	NaS_6/YS_6	$NaF_9/(Na/Y)F_9$
Minimum cation distance (Y^{3+}-Y^{3+})	3.9808	3.6379
Covalence	Strong (electronegativity of S is 2.58)	Weak (electronegativity of F is 3.98)
Phonon energy	278 cm^{-1}	350 cm^{-1}

Supplementary Table 2 Decay time constants τ (ms) of $^4S_{3/2}\rightarrow{}^4I_{15/2}$ (564 nm), $^4F_{9/2}\rightarrow{}^4I_{15/2}$ (650 nm), $^4I_{9/2}\rightarrow{}^4I_{15/2}$ (800 nm), $^4I_{11/2}\rightarrow{}^4I_{15/2}$ (1000 nm), and $^4I_{13/2}\rightarrow{}^4I_{15/2}$ (1500 nm) transitions in $NaYS_2$:xEr^{3+} (x=2, 5, 10, 15 mol%).

Samples	$^4S_{3/2}$	$^4F_{9/2}$	$^4I_{9/2}$	$^4I_{11/2}$	$^4I_{13/2}$
$NaYS_2$:2%Er^{3+}	0.87	1.82	10.28	2.33	33.04
$NaYS_2$:5%Er^{3+}	3.37	5.78	9.24	2.18	30.27
$NaYS_2$:10%Er^{3+}	3.25	6.94	6.18	1.94	19.18
$NaYS_2$:15%Er^{3+}	2.24	5.44	4.80	1.67	9.44

Supplementary Table 3 Decay time constants τ (ms) of different transitions for Er^{3+} in $MLnS_2$:Er^{3+} under 1532 nm excitation and $NaYF_4$:Yb^{3+},Er^{3+} under 980 nm excitation.

Samples	$^4S_{3/2}$	$^4F_{9/2}$	$^4I_{9/2}$	$^4I_{11/2}$	$^4I_{13/2}$
α-$LiYS_2$	1.26	2.74	2.40	2.83	4.69
β-KYS_2	2.37	3.87	7.45	2.54	13.12
α-$NaLaS_2$	1.77	3.05	2.84	2.52	7.08
β-$NaYS_2$	3.37	5.78	9.24	2.18	30.27
β-$NaLuS_2$	2.47	3.76	9.03	1.97	29.28
β-$NaGdS_2$	1.64	3.42	7.78	1.41	18.77
β-$NaYF_4$	0.58	0.45	0.98	--	9.92

Supplementary Table 4 Decay times of $^4I_{13/2}$, $^4I_{9/2}$, and $^4S_{3/2}$ in $NaYS_2:Er^{3+}$ under 980 nm and 1532 nm at low tempera (10 K) and room temperature

	10 K (1532 nm)	300 K (1532 nm)	10 K (980 nm)	300 K (980 nm)
$^4I_{13/2}$	15.3 ms	12.81 ms	32.5 ms	30.27 ms
$^4I_{9/2}$	22.55 ms	9.24 ms	39.5 ms	12.99 ms
$^4S_{3/2}$	6.32 ms	3.37 ms	--	--

Supplementary Table 5 A list of W and $W(0)$ for $^4S_{3/2}$, $^4F_{9/2}$, $^4I_{9/2}$ levels in $NaYS_2:Er^{3+}$ and $NaYF_4:Yb^{3+},Er^{3+}$.

		$^4S_{3/2}$	$^4F_{9/2}$	$^4I_{9/2}$	Ref.
$NaYS_2:Er^{3+}$	W/ms^{-1}	0.087	0.064	0.025	This work
	$W(0)$/ms^{-1}	0.011	0.014	0.006	
$NaYF_4:Yb^{3+},Er^{3+}$	W/ms^{-1}	1.01	1.24	--	[2]
	$W(0)$/ms^{-1}	0.08	0.90	--	

Supplementary Table 6 Performance of several NIR photodetectors

Material and structure	Spectral range [nm]		Responsivity [A W^{-1}]	Detectivity [Jones]	EQE	Response Time [ms]	Ref.
Commercial Ge	800-1800		0.85	3.0×10^{11}	--	--	[4]
Graphene/Si	850-900		0.44	7.7×10^{9}	--	> 1	[5]
PbS quantum dots	400-1500		--	--	--	350	[6]
Cu_2SnS_3 quantum dots	1550		0.93×10^{-3}	--	--	410	[7]
SiO_2 based BSR-PhOFETs	405, 532, 655, 850	850	0.58	1.5×10^{10}	85	--	[8]
Black phosphorus	1550		6.0×10^{-3}	--	-	0.1	[9]
UCNPs/graphene	808		4.0	--	-	--	[10]
UCNPs/graphene/GaAs	980		5.97×10^{-3}	1.1×10^{11}	-	--	[11]
Graphene nanoribbon	1550		1.0	--	--	--	[12]
DPPTT-T/PC_70BM/UCNPs	1550		0.73×10^{-3}	--	--	0.08	[13]
UCNPs/PDPP4T	NIR	808	0.56	--	86	--	[14]
		975	1.42	--	180	--	
		1532	0.02	--	2	--	
$NaYS_2$: Er/$MAPbI_3$	NIR	808	0.26	4.6×10^{10}	39	567	This work
		980	0.44	5.6×10^{10}	55	515	
		1550	0.73	8.4×10^{10}	59	480	

Supplementary References

[1] Boyer, J. C. & van Veggel, F. C. J. M. Absolute quantum yield measurements of colloidal $NaYF_4$:Er^{3+},Yb^{3+} upconverting nanoparticles. Nanoscale **2**, 1417-1419 (2010).

[2] Yu, W. Xu, W. Song, H. W. & Zhang, S. Temperature-dependent upconversion luminescence and dynamics of $NaYF_4$:Yb^{3+}/Er^{3+} nanocrystals: influence of particle size and crystalline phase. Dalton Trans. **43**, 6139-6147 (2014).

[3] Lin T. & Wang J. Z. Strategies toward high-performance s-processed lateral photodetectors. Adv. Mater. **31**, 1901473 (2019).

[4] Saran, R. & Curry, R. J. Lead sulphide nanocrystal photodetector technologies. Nat. Photonics **10**, 81-92 (2016).

[5] An, X. H. Liu, F. Z. Jung, Y. J. & Kar, S. Tunable graphene-silicon heterojunctions for ultrasensitive photodetection. Nano Lett. **13**, 909-916 (2013).

[6] Ageno, W. Eikelboom, J. & Lip. G. Y. H. Dabigatran in clinical practice: contemporary overview of the evidence. Int. J. Cardiol. **220**, 417-428 (2016).

[7] Dias, S. Kumawat, K. Biswas, S. & Krupanidhi, S. B. Solvothermal synthesis of Cu_2SnS_3 quantum dots and their application in near-infrared photodetectors. Inorg. Chem. **56**, 2198-2203 (2017).

[8] Huang, F. B. et al. Towards high performance broad spectral response fullerene based photosensitive organic field-effect transistors with tricomponent bulk heterojunctions. Carbon **118**, 666-674 (2017).

[9] Na, J. Park, K. Kim, J. T. Choi, W. K. & Song, Y. W. Air-stable few-layer black phosphorus phototransistor for near-infrared detection. Nanotechnology **28**, 085201 (2017).

[10] Kataria, M. et al. Transparent, wearable, broad band, and highly sensitive upconversion nanoparticles and graphene based hybrid photodetectors. ACS Photonics **5**, 2336-2347 (2018).

[11] Wu, J. H. et al. Enhanced performance of a graphene/GaAs self-driven near-infrared photodetector with upconversion nanoparticles. Nanoscale **10**, 8023-8030 (2018).

[12] Chitara, B. Panchakarla, L. S. Krupanidhi, S. B. & Rao, C. N. R. Infrared photodetectors based on reduced graphene oxide and graphene nanoribbons. Adv. Mater. **23**, 5419-5424 (2011).

[13] Xiang, H. Y. et al. Heavy-metal-free flexible hybrid polymer-nanocrystal photodetectors sensitive to 1.5 μm wavelength. ACS Appl. Mater. Inter. **11**, 42571-42579 (2019).

[14] Zhao, X Y. Song, L. Zhao, R. & Tan, M. C. High-performance and flexible Shortwave infrared photodetectors using composites of rare earth-doped nanoparticles. ACS Appl. Mater. Inter. **11**, 2344-2351 (2019).
