# Supplementary material for: Towards highly efficient NIR II response up-conversion phosphor enabled by long lifetimes of Er3+
Source: Nat Commun. 2022 Nov 1;13:6549. doi: 10.1038/s41467-022-34350-1 (PMC9626601; doi:10.1038/s41467-022-34350-1)
Supplement: Supplementary file 2 — Description of Additional Supplementary Files [file 41467_2022_34350_MOESM2_ESM.pdf]

File Name: Supplementary Data 1
Description: Source data of UCQYs.

File Name: Supplementary Movie 1
Description: Luminescence comparison for $NaYS_2:Er^{3+}$ excited at 1532 nm and $NaYF_4:Yb^{3+},Er^{3+}$ excited at 980 nm at same power density and photon flux.

File Name: Supplementary Movie 2
Description: Underwater information transmission application of $NaYS_2:Er^{3+}$.
